# Rho GTPase overexpression impacts leaf internal architecture and mesophyll conductance in Arabidopsis

**DOI:** 10.1111/nph.70618

**Published:** 2025-10-13

**Authors:** Isabella Østerlund, Silas Ørting, Alistair Leverett, Guillaume Théroux‐Rancourt, Samira Ebrahimi, Yang Wang, Zoran Nikoloski, Johannes Kromdijk, Staffan Persson

**Affiliations:** ^1^ Department of Plant and Environmental Sciences, Copenhagen Plant Science Center University of Copenhagen Frederiksberg C 1871 Denmark; ^2^ Bioinformatics Department Institute of Biochemistry and Biology, University of Potsdam Potsdam‐Golm 14476 Germany; ^3^ Department of Computer Science University of Copenhagen Copenhagen 2100 Denmark; ^4^ QIM—Center for Quantification of Imaging Data from MAX IV Copenhagen 2100 Denmark; ^5^ Department of Plant Sciences University of Cambridge Cambridge CB2 UK; ^6^ Department of Ecosystem Management, Climate and Biodiversity Institute of Botany, BOKU University Vienna 1180 Austria; ^7^ Niels Bohr Institute University of Copenhagen Copenhagen Ø 2100 Denmark; ^8^ Systems Biology and Mathematical Modeling Max Planck Institute of Molecular Plant Physiology Potsdam 14476 Germany; ^9^ Joint International Research Laboratory of Metabolic and Developmental Sciences, School of Life Sciences and Biotechnology Shanghai Jiao Tong University Minhang, Shanghai 200240 China

**Keywords:** air path tortuosity, air space, *Arabidopsis thaliana*, instance segmentation, leaf cell architecture, mesophyll conductance, persistent homology, semantic segmentation

## Abstract

- Leaves are built from multiple cell types and are structured to enable the conversion of carbon dioxide and water into sugars in the process of photosynthesis. Understanding how cell architecture impacts the movement of CO_2_ within leaves may provide means to improve photosynthesis. Here, we examined the impact of mesophyll cell architecture on air networks and air permeability by employing high‐resolution tomography data in leaves of *Arabidopsis thaliana* wild‐type plants and in plants with altered Rho of Plant (ROP)‐GTPase‐related activities.
- We employed high‐resolution tomography to characterise leaf cell architecture and associated air space networks. The image data were segmented and analysed using machine learning, and combined with leaf gas exchange measurements to evaluate photosynthesis‐related traits.
- We found that changes in the ROP‐GTPase pathway substantially altered the leaf cell architecture, causing disruptions in the air space network associated with higher tortuosity. In addition, changes in ROP‐GTPase activity resulted in reduced mesophyll conductance.
- Our observations underscore how changes in leaf cell architecture potentially drive alterations in photosynthesis‐related traits, highlighting a mechanistic link between mesophyll geometry, air space organisation, and CO_2_ diffusion.

## Introduction

The leaf of the model plant *Arabidopsis thaliana* (Meinke *et al*., 1998) is a complex, three‐dimensional laminar organ consisting of three main cell groups organised in layers. Photosynthetic mesophyll cells, sandwiched between adaxial and abaxial pavement cells, are further divided into densely packed palisade cells, optimised for light capture, above loosely packed spongy mesophyll cells with complex shapes. The complex shapes of these cells lead to intricate air spaces between them, which facilitate gas exchange (Kalve *et al*., 2014; Lehmeier *et al*., 2017; Borsuk *et al*., 2022). This arrangement is interspersed with a network of vascular tissue that transports water and nutrients through the leaf.

Both light harvesting and gas exchange are crucial for photosynthesis and depend on the organisation of mesophyll cells and the air spaces within the leaf (Evans, 2013). Disruptions of leaf cell architecture can affect mesophyll conductance of carbon‐dioxide (CO_2_) to chloroplasts and ultimately impair photosynthetic efficiency. A key problem, therefore, is to understand how mesophyll geometry impacts the uptake of air and light, an essential step towards revealing how altered leaf cell organisation affects photosynthesis (Taiz *et al*., 2023).

The spongy mesophyll cells form a porous tissue (Treado *et al*., 2022) that contains most of the internal air space of the leaf (Buckley *et al*., 2015). The air space, together with the spongy mesophyll cells, facilitates gas flow and helps cooling by transpiration (Whitewoods, 2021). Despite the porous structure of the internal parts of the leaf, cell–cell adhesions are able to maintain the structural integrity of the organ.

Therefore, the geometry of mesophyll cells dictates the shape of the internal air space around them, initially created through schizogeny, where cells detach from each other. During later developmental stages, the air space is enlarged by regional cell wall expansion adjacent to existing air spaces, known as expansigeny (Whitewoods, 2021; Zhang *et al*., 2021). The cytoskeleton of the plant cell regulates the deposition of cellulose fibres, which critically underpin cell wall strength and therefore control the direction of cell expansion and morphology (Brembu *et al*., 2006; Fletcher & Mullins, 2010). Small GTPases of the Rho of Plant (ROP) and ROP‐interactive CRIB‐containing proteins (RIC) regulate the cytoskeletal organisation, and this pathway underpins the puzzle piece‐like shapes of pavement cells (Fu *et al*., 2005; Sapala *et al*., 2018, 2019; Whitewoods, 2021). Indeed, overexpression of RIC1 or constitutively active ROP2 reduced the shape complexity of leaf pavement cells by impeding the lobe‐neck formation, which gives resemblance to puzzle pieces (Fu *et al*., 2005). The influence of GTPases on the development of spongy mesophyll cells is not yet fully understood. Nevertheless, the cytoskeleton is also important to mesophyll morphogenesis, and thus indirectly in the expansion of the internal air space of the leaf (Panteris & Galatis, 2005; Whitewoods, 2021). We therefore hypothesise that perturbing the cytoskeleton through the GTPase pathway also affects mesophyll cell architecture and consequently the internal air space, potentially influencing photosynthetic efficiency.

Leaf air space has gained renewed interest with recent advancements in imaging techniques. These techniques now enable studies to go beyond the first pavement cell layer and investigate internal leaf air space. Related studies have revealed relationships between mesophyll porosity and depth within the leaf (Lehmeier *et al*., 2017). For instance, Lundgren *et al*. (2019) observed a correlation between internal air space and stomatal patterning on the adaxial side of leaves. Earles *et al*. (2018) and Harwood *et al*. (2021) both investigated the tortuosity of air pathways, an indicator of gas flow efficiency, using different methods to highlight an inverse correlation between porosity and tortuosity.

Leaf anatomy has commonly been studied in two dimensions, resulting in biased estimation of the internal three‐dimensional (3D) properties, such as mesophyll cells (Théroux‐Rancourt *et al*., 2017; Harwood *et al*., 2020; Wolny *et al*., 2020). Recent advances in 3D imaging using X‐ray phase‐contrast micro‐tomography (X‐PCI) may address shortcomings of tissue sections that need chemical fixation and staining, which could alter cell morphology (Yu *et al*., 2021; Attuluri *et al*., 2022). However, accurate segmentation of X‐PCI images is challenging and a major limiting step in the analysis pipeline. To this end, several machine learning (ML)‐based methods have been developed for tissue segmentation, using, for example, Random Forests (Théroux‐Rancourt *et al*., 2020) or Convolutional Neural Networks (Rippner *et al*., 2022). Other ML and deep learning‐based methods have been developed for segmentation of single cells, such as Cellpose (Stringer *et al*., 2021), Cellpose 2.0 (Pachitariu & Stringer, 2022), and Stardist (Schmidt *et al*., 2018), as well as a method specifically developed for tightly packed plant cells (Wolny *et al*., 2020). However, none of these tools could accurately segment our 3D image data for leaf mesophyll tissue and particularly struggled with the more intricate shapes of the spongy mesophyll.

In this study, we investigate whether manipulating the GTPase pathway – using plants overexpressing either RIC1 (RIC) or constitutively active ROP2 (ROP) – alters the geometry of internal leaf cells in *Arabidopsis thaliana*. We further ask whether such alterations influence gas exchange and photosynthetic efficiency. To address these questions, we used high‐resolution 3D X‐PCI data that we segmented both at a tissue‐ and single‐cell level using deep learning with a human‐in‐the‐loop approach. We then analysed the organisation of spongy mesophyll cells and internal air space using persistent homology (PH) and examined the extent to which alterations in leaf cell architecture affected mesophyll conductance and photosynthetic efficiency.

## Materials and Methods

### Experimental setup for experiments

#### Plant material and experimental setup for X‐PCI

To study leaf development we use leaves from *Arabidopsis thaliana* Columbia‐0, CA‐ROP2 (Li *et al*., 2001) and 35S:RIC1 (Fu *et al*., 2005) lines at ages 3 wk (W3) and 5 wk (W5) from day of sowing. Seeds were sown in a 10‐cm‐diameter pot filled with a 5 : 2 potting soil : sand substrate mixture and stratified for 4 d at 4°C. Pots were then transferred to a growth cabinet and grown at 12 h : 12 h, 21.5°C : 18°C, day : night photoperiod with 60% relative humidity and ambient CO_2_. Pots were sown again 2 wk later for the younger plants. Three weeks later, the pots were brought to be imaged using Synchotron X‐PCI, at the TOmographic Microscopy and Coherent rAdiology experimentTs (TOMCAT) beamline of the Swiss Light Source at the Paul Scherrer Institute (Stampanoni *et al*., 2006).

We imaged sixth to eighth leaves counted from the first true leaf for both age groups. The leaves were first cut from the plant and an *c*. 1 × 1‐cm section was immediately cut from the lamina, avoiding the main vein and apparent higher order veins. To ensure reproducibility, we made sure that the same areas of the leaves were used across different leaves. The leaf pieces were then embedded into multiple layers of polyimide tape (Kapton; three to four layers on each side), the tape sealed tightly together using a razor blade and then cut into a small square by removing excess tape. When embedding the cut‐out, the Kapton tape should not touch the cut‐out, and the pocket of tape did not contain much air to avoid rotation artefacts. The leaf sample was then mounted into a polystyrene block fixed to a sample holder. Each scan was done using 1801 projections of 100 ms at 21 keV and 20× magnification, with voxel size on 0.325 μm.

#### Plant material and experimental setup for gas exchange

Seeds were stratified at 4°C for 3 d, then transferred to a Fitotron controlled growth cabinet, and allowed to germinate. After 20 d, seedlings were transferred to 165‐ml pots, containing Levington™ Advance F2 compost. Plants were watered once a week for 45 d and then twice a week for the remainder of the experiment. Because the Licor gas exchange cuvettes require a larger leaf, plants were grown under short day conditions (9 h : 15 h, light : dark) to maximise leaf area. Light : dark temperature was 21°C : 18°C and relative humidity was maintained at 50/60%. Gas exchange was conducted between Day 68 and 75 on Leaves 11–13.

### Plant material and experimental setup for leaf size and stomata measurements

The plants were grown under 12 h : 12 h day : night condition, light intensity 150 μmol ms^−1^, at the temperature of 21.5°C : 18°C. After germination, plants were transferred to 10‐cm‐diameter pots with four plants to each pot. From the second to sixth week after transferring, the sixth to eighth leaves counted from the first true leaf were cut from the plants and imaged under Keyence VHX7000 microscope. Images were segmented and leaf sizes were extracted.

### Image analysis

For computational reasons, all images were down‐sampled by a factor of four before segmentation and analysis. Although the images have a rectangular field of view, it is only the data within the enclosed maximal cylinder that were reconstructed from a full set of projections. We refer to this as the ‘valid cylinder’. Data outside the valid cylinder are of lower quality with more artefacts and is excluded in analyses. It is included in the segmentation process because it provides a more informative boundary than if it is removed.

#### Segmentation

Each image was segmented into six classes: mesophyll, outside leaf, veins, adaxial pavement, abaxial pavement, and internal air. The mesophyll class was further segmented into a palisade class and a spongy class. The palisade class consisted of the mesophyll cell layer closest to the adaxial pavement cells. The spongy class consisted of the two mesophyll cell layers closest to the abaxial pavement cells. The palisade class was segmented into individual cells for size measurements.

We used a combination of neural networks and morphology for segmentation. Details are provided in the Supporting Information Notes S1. The networks were trained from scratch using an iterative approach. A small number of annotations were used to train a network. The predictions of the trained network were then inspected, and a small number of predictions corrected. The corrected predictions were then added to the training data, and the network was retrained on the expanded training data. This process was iterated until no further improvement was observed by visual inspection of all volumes.

#### Air path tortuosity

For the analysis of air path tortuosity (*τ*), we computed the shortest distance from the abaxial pavement within the airspace to the adaxial pavement boundary exposed to internal air. This is different from previous methods used to characterise tortuosity in plant leaves (e.g. Earles *et al*., 2018) where tortuosity was expressed as the median value measured from stomata to the diffusive surfaces of mesophyll cells. For our method, we used $\tau \left(p,S\right)=d_{\mathrm{air}}\left(p,S\right)/d_{\text{direct}}\left(p,S\right)$, where $d_{\text{direct}}\left(p,S\right)$ is the shortest distance from a point *p*, the abaxial surface, to a surface *S*, the adaxial surface, and $d_{\mathrm{air}}\left(p,S\right)$ the shortest distance between those points through the internal airspace. We used the fast marching method (FMM) to compute both *d*_direct_ and *d*_air_. This is defined as FMM Tortuosity (Holzer *et al*., 2023).

#### Palisade cell length

We defined palisade cell length as the maximum side length of the bounding box of the cell. This is also known as the Feret diameter. To measure the Feret diameter, we first rotate each cell such that the inertia tensor of the cell is aligned with the image grid and then measure the maximal side length of the bounding box. We exclude all cells fully or partially outside the valid cylinder and exclude all cells that do not touch the top epidermis layer. This is to ensure that only complete palisade cells are measured.

#### Tissue group analyses

**Fig. 1:**
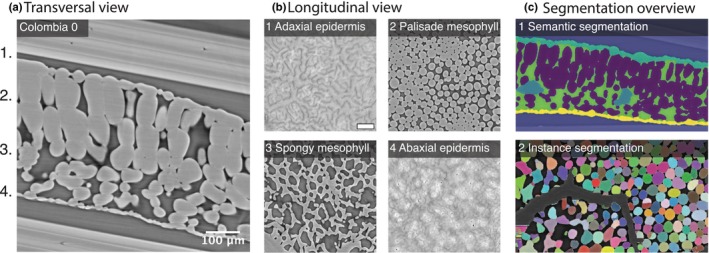
Cell structure and segmentation overview of Columbia‐0 (Col‐0) *Arabidopsis thaliana* leaf. (a) Illustration of X‐ray phase‐contrast micro‐tomography image of the transversal view of 5‐wk‐old wild‐type (WT) leaf scan. (b1–b4) illustrate longitudinal views of the four different cell layers from the same image stack as portrayed in (a): (1) adaxial epidermis; (2) palisade mesophyll; (3) spongy mesophyll; and (4) abaxial epidermis layer. Bar, 100 μm. (c) Illustrative overview of segmentation methods. (c1) illustrates 2D representation of semantic tissue segmentation. Blue represents outside leaf, teal represents adaxial epidermis, yellow represents abaxial epidermis, green indicates internal air space, cyan the veins, and purple is illustrating the palisade and spongy mesophyll cells. (c2) illustrates 2D representation of an instance cell segmentation, where each defined cell has a unique colour.

From the semantic tissue segmentation, the raw image data were segmented into six groups, where five groups represent leaf tissue groups and the last one represents outside leaf (Fig. 1c1). These groups are used to calculate the ratio of which tissue group makes up a leaf. Each group of tissue contains its own unique identifier, which is used to sum the volume that each group covers. Each value is normalised to the full volume of the leaf, resulting in a ratio. The thickness of each leaf scan is found using a distance transformation of a mask covering the five groups that represent the leaf. The line of maximum value of the distance transformation of the mask represents the thickness of the leaf. This is performed on each slice of the 3D data, and the mean value is used as a final representation of the thickness. The value is multiplied by 2, since it is the centre that is found each time with the distance transformation. To calculate the bootstrapped SE, we bootstrapped 1000 times with a confidence level of 0.95 and then found the SD on the bootstrapped values.

These calculations were performed in python and were heavily dependent on scipy (Virtanen *et al*., 2020) and scikit‐image (Van Der Walt *et al*., 2014) packages.

#### Air to cell surface and 2D porosity profiles

For each 2D *x‐z* (transversal, *x*‐*y* corresponds to longitudinal view) slice in the 3D image data, air to cell surface is calculated. The air to cell surface is calculated as a 1D value, where the ‘free cell surface’ is defined by transforming the mask for air using signed Euclidean distance transformation (SEDT), and then, the ‘free cell surface’ is defined by the values equal to 0. For each 2D slice, this value is found and divided by the total surface of defined tissue in the same 2D slice, resulting in the air to cell surface profile moving from the abaxial to adaxial side of the leaf. This value is calculated for all slices, resulting in a gradient ratio of 2D air to surface as we go from the adaxial to abaxial side of each leaf scan.

The 2D porosity is calculated in a similar manner; the porosity (air to tissue ratio) for each slice in the *x*‐*z* plane is calculated, moving from the adaxial to abaxial side of the leaf.

#### Persistent homology analysis

To perform PH on the segmented 3D image data, we treated the data as a two‐phase system: air space and leaf tissue. The binarised image data were translated using a SEDT for both spaces, assigning negative values to air and positive values to tissue. Filtration was then applied to the SEDT data, starting from the most negative values and incrementally progressing to the most positive. This process identifies topological changes as we filter through the data, tracking the birth and death of features (known as Betti or *β*‐numbers), focusing on connected components and voids or cavities across different value ranges. Birth represents the appearance of a new feature, and death marks its disappearance, with the persistence of each feature defined by the difference between birth and death.

A persistence diagram, which graphically represents birth and death, can be divided into four quadrants: the first is the upper right (positive deaths and positive births), the second is upper left (positive death and negative birth), the third is lower left (negative death and negative birth), and lastly the lower right (negative death and positive birth). See Fig. S1 for an overview.

For the topological feature *β*_0_, representing air bodies, birth occurs in the negatively signed air space. These features die when they merge with another *β*_0_, either in the air space (negative death) or in the tissue space (positive death). A negative death indicates a connected air body, while a positive death indicates that it is a disconnected air body. The birth value informs the maximal enclosed air space radius. In the persistence diagram, the connected air bodies will lie in the third quadrant and the disconnected bodies will lie in the second quadrant. We will focus on the third quadrant of *β*_0_ features, informing on the varying size of the internal air space.

The *β*_1_ feature represents 1D loops and rings. Loops formed in tissue space appear in the first quadrant, redundant loops around tissue in air are in the second, and loops formed in air space reside in the third quadrant. However, this feature is not investigated here, as the analysis focuses on air and tissue body sizes.

The *β*_2_ feature corresponds to voids or concavities (spongy mesophyll). As filtration progresses, the cells become isolated from one another, with death values indicating individual cell radii. These features have positive deaths and births, and lie in the first quadrant of the persistence diagram. We will focus on the first quadrant for *β*_2_, showing the sizes of individual cells.

Points near the diagonal in persistence diagrams (*d* ≈ *b*) are considered to have low persistence. In this study, we removed features with a birth value smaller than one pixel to reduce noise. PH was calculated using the CubicalRipser Python library (Kaji *et al*., 2020), and the calculations are based on the work of Herring *et al*. (2019).

### Stomata density and size calculation for leaf size measurements

For each genotype, three leaves were sampled, using the seventh leaf from the bottom, using the same leaves as used in the leaf size experiment. Two sections of the middle part of the leaves were cut and stained with propidium iodide for abaxial and adaxial imaging, respectively. For each cut, two fields of view were imaged to alleviate sampling bias. Since the surfaces are not flat, the z‐stacks were taken with a 40× 0.95 air objective using the Marianas – 3i spinning disc microscope.

Roughly 8 to 15 slices under the surface of the Z‐stacks were projected as 2D images using the fiji plugin surfcut (Erguvan *et al*., 2019). Subsequently, stomata were hand segmented, and stomata sizes were computed with Napari (Chiu & Clack, 2022). The stomata density was calculated as the ratio between the number of stomata and the area of the field of view. The two fields of view correspond to two data points per leaf. The area of stomata was given by the hand segmented masks of stomata. Statistical analyses were performed with the linear mixed effect model, followed by the Tukey method for group comparisons.

### Physiological measurements for gas exchange experiments

Gas exchange and pulse amplitude modulated (PAM) fluorescence were measured concurrently using a Licor‐6800 (Licor, Lincoln, NE, USA). Net CO_2_ assimilation (*A*_*n*_) and CO_2_ concentration in the substomatal cavities (*c*_*i*_) were taken directly from the Licor‐6800 instrument. Stomatal conductance to CO_2_ (*g*_sc_) was estimated by dividing the stomatal conductance to water by 1.6, to account for the differences in diffusivities of the two gasses (Jarvis, 1971). Mesophyll conductance (*g*_m_) was estimated using the variable *J* method (Harley *et al*., 1992). All measurements were made under light intensity of 700 μmol m^−2^ s^−1^. Measurements were made at atmospheric CO_2_ concentrations (*c*_a_) of 410, 360, 310, and 410 μmol mol^−1^. Each time the atmospheric CO_2_ concentration (*c*_a_) was altered, leaves were given 25 min to acclimate before measuring. At each *c*_a_, the CO_2_ concentration in the chloroplast, *c*_c_, was estimated as done by Harley *et al*. (1992).

$$c_{c}=\frac{\Gamma^{*}\left(J+8\left(A_{n}+R_{L}\right)\right)}{J-4\left(A_{n}+R_{L}\right)}$$

where $\Gamma^{*}$ is the photorespiratory CO_2_ compensation point, *J* is the chloroplast electron transport rate, *A*_*n*_ is net CO_2_ uptake, and *R*_L_ is mitochondrial respiration rate in light. $\Gamma^{*}$ was assumed to be 42.5 μmol mol^−1^ (normal range: 40–45 μmol mol^−1^) or was estimated from *A*_*n*_/*c*_*i*_ curves according to the Laisk (1977). The Laisk curves were constructed by measuring *A*_*n*_ under a varying range of *c*_a_ concentrations, (25, 40, 60 and 80 μmol mol^−1^) under three sub‐saturating light intensities (110, 70, and 50 μmol m^−2^ s^−1^). *R*_L_ was taken as the y intercept using the method of Yin *et al*. (2009). The apparent photorespiratory CO_2_ compensation point ($c_{i}^{*}$) was estimated using the regression‐intercept approach defined by Walker and Ort (Walker & Ort, 2015), and $\Gamma^{*}$ was calculated according to

$$\Gamma^{*}=c_{i}^{*}\cdot \frac{R_{L}}{g_{m}}$$

Both $\Gamma^{*}$ and *g*_m_ are unknown, and where estimated by iterative fitting until a stable value was reached.

To estimate the chloroplast electron transport rate (*J*), gas exchange and PAM fluorescence were used in tandem, according to

$$J=Q_{\text{in}}\cdot s\cdot \frac{F_{m}^{\prime }-F^{\prime }}{F_{m}^{\prime }}$$

where $Q_{\text{in}}$ is the light intensity, $F^{\prime }$ is steady state fluorescence, and $F_{m}^{\prime }$ is maximal fluorescence. The correction factor, *s*, was estimated by taking the gradient between *A*_*n*_ and $Q_{\text{in}}\cdot \frac{F_{m}^{\prime }-F^{\prime }}{F_{m}^{\prime }}/4$, measured under 2% O_2_ and 1200 μmol mol^−1^, under a range of sub‐saturating light intensities (140, 120, 100, 80, and 60 μmol m^−2^ s^−1^) (Putten *et al*., 2018). When conducting the Yin method, leaves were allowed to acclimate for 20 min between each measurement. The intercept of this slope was used to estimate −*R*_L_, based on the assumption that *R*_L_ does not change with light intensity.

Once *c*_c_ had been estimated, *g*_m_ was determined according to Fick's first law of diffusion.

$$g_{m}=\frac{A_{n}}{c_{i}-c_{c}}$$

### Estimation of Maximal Rubisco carboxylation capacity

Maximal Rubisco carboxylation capacity, $V_{\text{cmax}}$, was estimated from the relationship between net CO_2_ uptake and the CO_2_ concentration in the chloroplast, by fitting

$$A=V_{\text{cmax}}\frac{c_{c}-\Gamma^{*}}{c_{c}+K_{c}\left(1+O/K_{o}\right)}-R_{L}$$

with $V_{\text{cmax}}$ as the slope and $R_{L}$ as the intercept. The Michaelis constant for carboxylation and oxygenation (*K*_c_ and *K*_o_ respectively) was taken from Boyd *et al*. (2019), using values estimated at 20°C, employing the radio labelling curve fitting method.

## Results

### Visualisation and quantification of leaf cell architecture in Arabidopsis leaves

To visualise and quantify the leaf cell architecture in Arabidopsis leaves, we used X‐PCI (Stampanoni *et al*., 2006) to image leaves of 3‐wk‐old (W3) and 5‐wk‐old (W5) Arabidopsis wild‐type (WT) plants (see the Materials and Methods section). We used these two developmental stages for two reasons: (1) to capture mature leaves with mesophyll cells of complex shapes, and (2) to compare against the RIC and ROP genotypes which grow in a similar pattern; however, they have smaller leaf areas than WT (Fig. S2). We scanned leaves at three locations along the leaf: close to the distal tip (top), mid‐part of the leaf (middle), and close to the base (bottom). To quantify leaf cell architecture, we first segmented on the tissue level (semantic segmentation assigns a class label to each pixel, thereby grouping together all pixels that belong to the same object category), and then on the single cell level (instance segmentation assigns an object ID to each pixel in the same class label, enabling the distinction between individual objects of the same class) from the raw tomography data. The segmentation models are explained in Notes S1. Each 3D leaf image stack was segmented into six main groups, namely outside the leaf, mesophyll cells, adaxial and abaxial pavement cells, vascular bundles, and air spaces (Fig. 1c1, cell type overview a, b, c1), resulting in six semantic segmentation groups. Individual mesophyll cells were also segmented (Fig. 1c2), resulting in instance cell segmentation based on the semantic segmentation of the mesophyll group. The leaf cell architecture and air spaces resulting from the segmentation were then quantified.

We found that leaves of W5 WT plants were thicker than those of W3 plants (Fig. S3A). These changes were largely driven by elongation of palisade cells (Fig. S4). By calculating the internal air to cell surface ratio (given as 1D free cell surface inside the leaves to 2D cell area per image slice), we found that more cell surface was exposed to air in W3 than in W5 leaves (Welch *t*‐test *P*‐val < 0.05). This change was due to the elongation of palisade cells, which became more densely packed over development, leading to a reduction in cell surface exposed to air (Fig. S3D). However, the ratio of mesophyll and air volume to total leaf volume did not change between the developmental stages (Fig. S3G,J). In addition, the pavement cells did not differ in thickness (transversal view) between developmental stages (Fig. S5).

### Persistent homology estimates reveal changes in internal air spaces of leaves

**Fig. 2:**
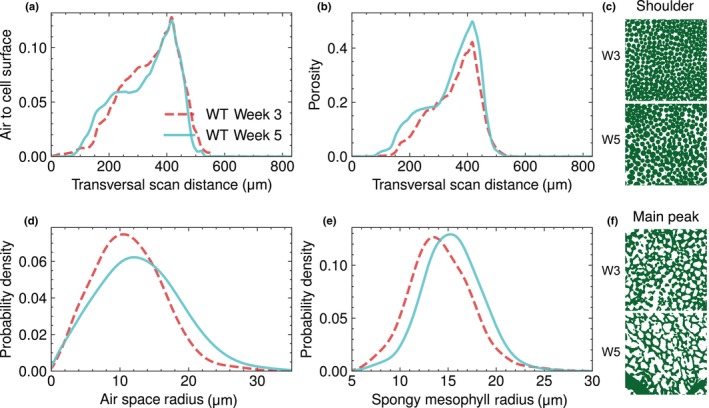
Developmental changes in the leaf cell architecture of maturing *Arabidopsis thaliana* leaves. (a) Air to cell surface ratio calculated from 2D slices starting from the adaxial to abaxial side of the leaf, or transversal scan distance. (b) Porosity calculated similarly to a, from 2D slices following transversal scan distance. Transversal scan distance reefers to the full depth of a scan in the transversal view, values containing zero are either edges of the leaves, or 2D slices not containing leaf data. (c) Tissue representation of palisade cells in leaves of week 3 (W3) and week 5 (W5) wild‐type (WT) plants, from the shoulder peak of the porosity distribution. (d) Kernel density estimation (KDE) histograms of air space radii around the spongy mesophyll cells. (e) KDE histograms of spongy mesophyll radii. Both (d, e) Plots are obtained using persistent homology calculations of the two first spongy mesophyll cell layers from the abaxial side of the leaves. (d) is portraying air space radii, and (e) is showing individual spongy mesophyll cell radii. Red illustrates W3, and turquoise illustrates W5 WT. (f) Tissue representation of spongy mesophyll cells in leaves of W3 and W5 WT plants, at the main peak of the porosity distribution.

We next investigated airspace changes as a function of depth through the leaves in the transversal scan view. The distribution of air to cell surface ratios around the spongy mesophyll cells was similar between leaves of W3 and W5 plants (Fig. 2a; Table S1; WT W3 mean: 0.13, WT W5 mean: 0.13, Welch's *t*‐test *P*‐val: 0.6). Porosity (air to tissue ratio 2D profiles, see the Materials and Methods section) revealed an increase (mean 0.42 to 0.50, Welch's *t*‐test *P*‐val: 0.0002) in air space from W3 to W5 plants in the spongy mesophyll region (Fig. 2b,f; Table S2 for visual confirmation), indicating that the proportion of air space surrounding spongy mesophyll cells increased slightly during development.

To further quantify the corresponding structural changes in the spongy mesophyll, we employed PH on two spongy mesophyll cell layers from the abaxial side. PH is a topological method used to analyse structures in 3D data by identifying and tracking features – such as voids, or connected components –across spatial scales (Robins *et al*., 2016; Herring *et al*., 2019; Sørensen *et al*., 2022).

In this context, homology refers to the classification of features based on their dimensionality (we focus on connected components and voids), while persistence quantifies how long each feature exists across a range of resolutions. This allowed us to distinguish between short‐lived noise and robust, biological structures. Using PH, we calculated radii for all bodies of air space, as well as cell radii for the spongy mesophyll cells. Here, the radius refers to the persistence of the feature, from the widest part of the structure before it merges or disappears. For example, for lobed spongy mesophyll cells, this corresponds to the cell centre that may connect to other cells to form a persistent connected component. For a complete overview of PH, refer to the Materials and Methods section.

We hypothesised that the alteration in porosity was caused by geometrical changes to the air space around the spongy mesophyll cells. PH analyses, however, did not show any significant differences in the modes of air space radius between W3 and W5 leaves (modes 3 wk: 9.82 μm, 5 wk: 11.38 μm, Welch's *t*‐test *P*‐val: 0.17; Fig. 2d). However, the distribution of air space radii differed significantly between developmental stages, as shown by the Kolmogorov–Smirnov (KS) test on the cumulative distributions (*P* = 0.0018). The spongy mesophyll cell radii increased slightly in leaves from W5 plants, compared with W3 plants (modes 3 wk: 14.04, 5 wk: 15.41), with a marginal difference in peak values (Welch's *t*‐test *P*‐val: 0.090), but a significant shift in distribution (KS test on cumulative distribution *P*‐val: 0.0021; Fig. 2e). Our findings indicate that the cell surface exposed to air is similar in W3 and W5 leaves, since the 2D ratio of air to cell surface remained similar in the region of spongy mesophyll cells across the two developmental stages.

### The transgenic lines 35S:RIC1 and CA‐ROP2 show altered leaf cell architecture

Given the pavement cell morphology defects in ROP and RIC (see Fig. S6 for pavement cell overview), we hypothesised that the transgenic lines may also have cell morphology defects inside the leaf, since similar pathways may also control cell shapes of spongy mesophyll cells (Panteris & Galatis, 2005; Whitewoods, 2021), which in turn may lead to altered tissue structure of the leaves.

To inspect whether the ROP and RIC plants grew similar to WT, we measured leaf areas across leaf development. We applied a two‐step threshold‐based change detection to assert plateauing growth (median leaf area growth < 10%). Our leaf area growth experiment showed that RIC and WT followed a similar trend in leaf expansion over time, with leaf growth plateauing after *c*. 4 wk (sixth to eighth leaf measured, WT median week: 4.3, RIC median week: 4.7, ROP median week: 5.0). Hence, ROP showed a slightly divergent growth pattern with continuous leaf growth between W4 and W5 (Fig. S2). The leaf areas were not expected to be the same, as the ROP and RIC transgenic lines have altered pavement cell shape, which limits leaf expansion (see Fig. S6 for pavement overview). Nevertheless, based on the growth behaviour, we chose to compare leaves from the three genotypes at W3 and W5.

We first observed that both transgenic lines grew less in the transversal view (thickness of the leaf) compared with WT (Fig. S3A–C). However, we observed a slight increase in the length of the palisade cells for ROP, but not for RIC (Figs S7, S8; Table S3). This is consistent with our findings regarding the growth of the transgenic lines, with ROP, but not RIC, showing a slight increase in leaf thickness between developmental stages (Fig. S3B,C). We further found a general change in pavement thickness between ROP and WT, but not for RIC (Fig. S9). However, there were no developmental differences in pavement thickness for a given genotype (Fig. S5).

**Fig. 3:**
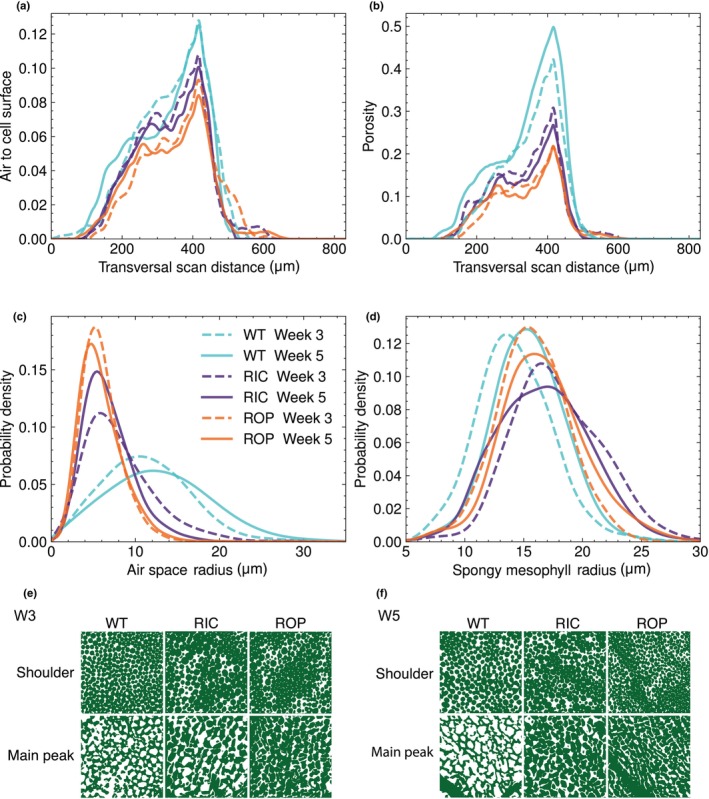
RIC and Rho of Plant (ROP) drive changes in cell morphology and air space architecture in *Arabidopsis thaliana* leaves. (a) Air to cell surface ratio calculated from 2D slices starting from the adaxial to abaxial side of the leaf, or transversal scan distance. (b) Porosity calculated similarly to (a), from 2D slices following transversal scan distance. Transversal scan distance refers to the full depth of a scan in the transversal view, values containing zero are either edges of the leaves, or 2D slices not containing leaf data. (c) Kernel density estimation (KDE) histograms of air space radii around the spongy mesophyll cells. (d) KDE histograms of spongy mesophyll radii. Both (c, d) Plots are calculated based on persistent homology calculations of the two first spongy mesophyll cell layers from the abaxial side of the leaves. (c, d) illustrate air space radii and individual spongy mesophyll cell radii, respectively. Turquoise illustrates wild‐type (WT), purple illustrates RIC and orange illustrates ROP. Broken lines illustrate W3 plant lines, and full lines denote W5 plant lines. (e) Tissue representation of palisade cells in leaves of week 3 (W3) and week 5 (W5) WT, RIC, and ROP plants, from the shoulder peak of the porosity distribution. (f) Tissue representation of spongy mesophyll cells in leaves of W3 and W5 WT, RIC, and ROP plants, at the main peak of the porosity distribution. RIC, ROP‐interactive CRIB‐containing proteins.

We next assessed free cell surface and 2D porosity as a function of depth in the transversal scan view in the different lines, see Materials and Methods section for more detail. Both RIC and ROP displayed consistently lower values of air to cell surface ratio as compared to WT in the main peak (with all Welch's *t*‐test *P*‐val < 0.05 between both W3 and W5 WT and RIC and ROP), corresponding to the region of spongy mesophyll cells (Fig. 3a,e,f). This indicates that spongy mesophyll cells in transgenic lines grow more spherically than those of WT. We also noted a general drop in porosity for the two transgenic lines (Fig. 3b), although the main peak still occurred in the spongy mesophyll cell layers. Both the 2D free cell surface and porosity values were lower in ROP than in RIC at the peak maxima across the leaves (Tables S1, S2), indicating a further suppressed ability of this line to form air space.

To further investigate the changes in cellular architecture in the spongy mesophyll tissue between the investigated lines, we employed PH on two spongy mesophyll cell layers from the abaxial side of the leaves. This was performed to investigate whether the changes in porosity and air to cell surface were caused due to geometrical changes in the spongy mesophyll tissue. We found that the peak in air space radius showed marginal differences between RIC and ROP (Welch's *t*‐test for W3 RIC and ROP *P*‐val: 0.098, W5 RIC and ROP *P*‐val: 0.038), but that the values were substantially lower than WT (Fig. 3c; Table S4; KS test on cumulative distributions RIC ages *P*‐val: 0.13, ROP ages *P*‐val: 0.998). Surprisingly, the peak values for spongy mesophyll cell radii were more similar across lines than those for air space radii (Fig. 3d; Table S4). While a few comparisons were statistically significant (e.g. W3 WT vs W3 RIC, Welch's *t*‐test *P*‐val: 0.006), most differences in cell radius across lines and timepoints were modest and not statistically significant. By contrast, air space radius distributions varied substantially between lines, reflecting greater divergence in air space geometry (Table S5). This is consistent with the finding that the internal air volume to tissue ratio is in general lower for RIC and ROP than for WT (air to tissue ratio: WT: 0.25 ± 0.004, ROP: 0.13 ± 0.003, RIC: 0.16 ± 0.004).

We therefore conclude that the spongy mesophyll cells in the two transgenic lines do not form the same lobe‐neck patterns as WT. Instead, they appear as spheroids, maintaining a slightly enlarged cell radii compared with WT spongy mesophyll cells (Fig. 3e,f, main peak; Fig. S10). In contrast to the WT, the spheroid shaped mesophyll cells thus create a denser tissue with an airspace formed by substantially smaller pores than WT. The lobe‐neck formation is important for air space creation and suggests a reduced ability of the cells in the RIC and ROP lines to undergo expansigeny growth.

### Air path tortuosity is associated with changes in the internal air space

Based on our findings about altered air space structures in the transgenic lines, we investigated whether air needs to travel relatively longer distances in a leaf of RIC and ROP compared with WT. To this end, we calculated the air path tortuosity, defined as the shortest path created by the internal air space, starting and ending in the two epidermal cell layers, normalised by the Euclidean distance. Therefore, tortuosity of one indicates that the path traversed by air is straight, and values larger than one indicate deviations from this shortest path. Refer to the Materials and Methods section for calculations of tortuosity.

**Fig. 4:**
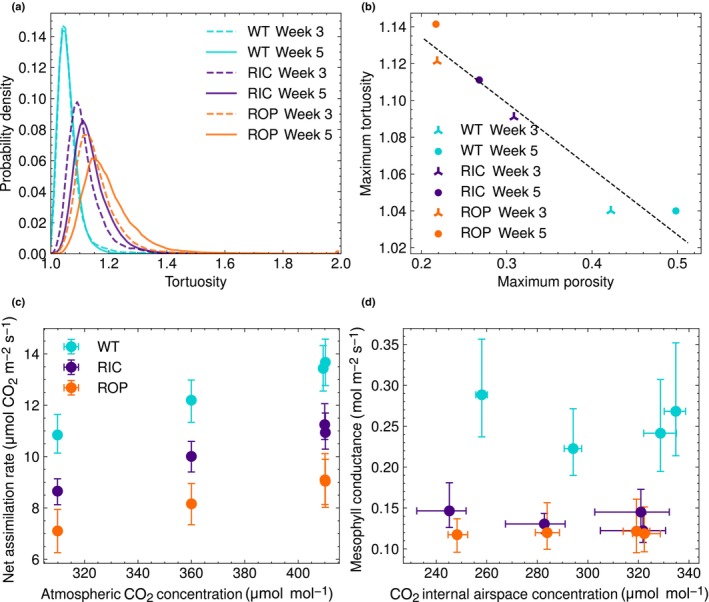
RIC and Rho of Plant (ROP) showcase altered air diffusion and mesophyll conductance. (a) The tortuosity probability density, which is the shortest path normalised by the Euclidean distance from the inside the abaxial pavement layer to the inside the adaxial pavement layer for each type and age. (b) The maximum tortuosity against maximum porosity for each plant type and age. Linear regression indicates a negative relation between maximum tortuosity and porosity. (c) Net assimilation rate against atmospheric carbon dioxide concentrations. (d) Mesophyll conductance against carbon dioxide concentration in internal airspace of the leaves. (c, d) illustrate with 1000 repetitions bootstrapped 90% confidence interval. RIC, ROP‐interactive CRIB‐containing proteins.

The tortuosity for WT was close to one, indicating that the cellular architecture is close to optimal in terms of air transport from abaxial to adaxial pavement cells. Furthermore, the tortuosity profiles for leaves of W3 and W5 WT plants were similar, showing that as a leaf grows in thickness, the air structure maintains an optimum. The tortuosity distributions were all statistically different between leaves of different ages (K‐S test *P*‐val < 0.05 for all three plant lines). The maximum tortuosity values for leaves of W3 RIC and ROP plants were greater than WT, and became increasingly higher over development (Table S6). The ROP leaves displayed the highest maximum tortuosity (Fig. 4a; W3: 1.12, W5: 1.14). This illustrates a sub‐optimal air space architecture, since air has to traverse longer to reach deeper cell layers for the two transgenic lines.

We furthermore found a negative correlation between maximum porosity and maximum tortuosity across all lines (Pearson correlation coefficient = −0.97, *P*‐val = 0.0017; Fig. 4b). Such negative correlation between porosity and tortuosity has been shown in other porous media (e.g. battery, soil and cement) and also in some C_3_ plants, where it is hypothesised that lower leaf porosity is related to lower exposure of mesophyll to internal air, which would lead to higher tortuosity (Earles *et al*., 2018).

Stomata in adaxial and abaxial epidermis are known to be coupled to large air pockets in the spongy mesophyll layer, where air spaces develop downstream of stomata, linking stomatal density and air space volume (Lundgren *et al*., 2019; Baillie & Fleming, 2020; Whitewoods, 2021). Therefore, we investigated whether the RIC and ROP lines showed any changes in stomatal density and size. These calculations were conducted on both adaxial and abaxial epidermis of leaves of WT and the two transgenic lines for synchrotron segmented data, photosynthetic experiments and an additional data set created to measure leaf size and stomata. Each data set was tested for normality using the Shapiro–Wilk test, all following a normal distribution. A one‐way ANOVA was performed, followed by Tukey's *post hoc* tests for pairwise comparisons.

On the abaxial side, we found no significant difference in stomatal density between WT and RIC. However, ROP exhibited significantly higher stomatal density than WT (*P* = 0.004; Table S7, based on synchrotron data from W5 plants, normalised to scanned leaf surface). This pattern was consistent in 10‐wk‐old leaves used for photosynthetic measurements (Table S8). The data set for leaf size and stomatal characteristics did not show significant differences in abaxial stomata density between any genotype (Fig. S11). On the adaxial side, stomatal density did not differ significantly between WT and RIC, but was significantly higher in ROP than in WT (*P* = 0.006; Table S9). This trend was also observed in the data set for leaf size and stomatal characteristics for W5 leaves. However, in W3 leaves, we did not detect a significant difference between WT and ROP; instead, RIC had the highest adaxial stomatal density (Fig. S11). In terms of stomatal size, both transgenic lines had larger stomata than WT, with ROP showing the largest stomata overall. These measurements were obtained for W5 plants on the adaxial side (Table S9) and for 10‐wk‐old leaves on the abaxial surface (Table S8). In the data set for leaf size and stomatal characteristics, adaxial stomatal size in W3 leaves did not differ significantly between genotypes. However, on the abaxial side of W3 leaves, WT had larger stomata than both RIC and ROP. For W5 leaves, the stomatal size was consistently larger in RIC and ROP than in WT on both adaxial and abaxial sides, in agreement with the other data (Figs S11, S12).

Interestingly, with higher stomatal density, one would perhaps expect higher airspace porosity in ROP to WT lines. Since we see opposite results, we conclude that the ectopic expression of RIC and ROP are likely directly affecting the cell shape, and that this is not an indirect consequence of altered stomatal density or anatomy.

### Alterations in internal airspace of RIC and ROP affect photosynthetic properties

Based on the findings that the transgenic RIC and ROP lines had altered cellular architecture and changed tortuosity values compared with WT, we next aimed to determine whether these lines differed in steady‐state photosynthetic assimilation rates in leaves, and to assess whether any differences are explained by increased internal diffusion resistances to CO_2_. The photosynthetic assimilation rates were investigated using gas exchange measurements.

Net assimilation rate of CO_2_ (*A*_*n*_) was measured at three atmospheric CO_2_ concentrations (*c*_a_) (*c*_a_: 310, 360, and 410 ppm), with an additional replicate measurement at 410 ppm taken at the end of the experimental protocol.

Net photosynthetic assimilation rate (*A*_*n*_) was consistently lower in both transgenic lines across all *c*_a_ values compared with WT (Fig. 4c). This indicates that the altered tissue architecture in RIC and ROP leaves is associated with reduced carbon assimilation under steady‐state conditions. To assess whether these reductions were statistically significant, we constructed an ANCOVA model with genotype and *c*_a_ as factors and *A*_*n*_ as the response variable (excluding the second 410 ppm replicate). The model revealed significant effects of both genotype and *c*_a_ on *A*_*n*_, with *post hoc* Tukey–Kramer tests confirming significant differences between all genotype pairs (*P* < 0.001 for all comparisons).

Furthermore, mesophyll conductance (*g*_m_) was lower in both transgenic lines than in WT plants (Fig. 4d). A second ANCOVA with genotype and intercellular CO_2_ concentration (*c*_*i*_) as predictors, and *g*_m_ as the response variable, showed that genotype, but not *c*_*i*_, had a significant effect on *g*_m_ (*P* < 0.001 and *P* = 0.1, respectively). Again, all pairwise genotype comparisons were significant in *post hoc* analysis. These trends held regardless of whether the photorespiratory compensation point ($\Gamma^{*}$) was assumed equal across lines or estimated from Laisk curve‐derived values (Figs S13, S14, S15; Notes S2). In addition, both RIC and ROP lines showed lower stomatal conductance (*g*_sc_). Finally, ROP plants exhibited significantly reduced CO_2_ carboxylation capacity (*V*_cmax.cc_), compared with WT plants (Dunn's test *P*‐val: 0.03), although no such difference was observed in RIC plants (Dunn's test *P*‐val: 0.73; Fig. S16).

Taken together, these results show that overexpression of RIC1 or constitutively active ROP2 leads to reduced photosynthetic capacity, in part through decreased mesophyll conductance.

## Discussion

In this study, we investigated the changes in leaf cell architecture across two developmental stages using X‐PCI imaging, deep learning‐based segmentation, and PH analyses. The use of deep learning allowed us to efficiently and accurately segment complex, high‐resolution image data into six main tissue groups, providing accurate masks for further analysis. PH is well suited for analysing the intricate geometrical structures of the porous spongy mesophyll due to the ability of this approach to capture topological features that are scale‐invariant and shape‐sensitive. PH has been employed for topological data analysis in various contexts, such as 3D x‐ray computed tomography of rock structures (Robins *et al*., 2016; Herring *et al*., 2019), and in biology to analyse plant branching structures and classify 2D leaf shapes (Li *et al*., 2017, 2018), but not for leaf cell shape analysis. Here, PH enabled a comprehensive examination of the cell and airspace architecture, which traditional methods might not capture.

Previous studies have tracked cell growth, focusing on the outer pavement cell layer or the first to second cell layers in a leaf (Willis *et al*., 2016; Zhang *et al*., 2021; Li *et al*., 2024). By contrast, our approach, combining X‐PCI imaging, deep learning based segmentation, and PH analyses, enabled investigation of morphological changes across all cell types in the leaf. Additionally, we were able to link these changes in cell architecture with alterations in gas exchange in the transgenic RIC and ROP lines, highlighting the significance of structural geometry for photosynthesis.

When comparing WT with transgenic lines, we observed that the spongy mesophyll cells in the latter appeared less complex in shape, leading to alterations in the internal air space. The transgenic lines exhibited substantially smaller air space radii than WT, which we further analysed using tortuosity. This analysis revealed a reduced capacity for air to penetrate deeper cell layers in the transgenic lines, showing a negative correlation between maximum porosity and tortuosity. Given the function of RIC and ROP, our findings suggest that the cytoskeleton plays a crucial role in shaping spongy mesophyll cells, likely by controlling anisotropic growth, which indirectly influences the properties of the leaf's air space. This aligns with a previously hypothesised mechanism by Panteris and Galatis for shaping both pavement and spongy mesophyll cells (Panteris & Galatis, 2005).

Through further investigation of gas exchange, we found that the transgenic lines exhibited reduced mesophyll conductance compared with WT, which led to lower steady‐state photosynthetic rates. These findings correlated well with our results from the X‐PCI data on altered internal airspaces, showing that the less complex shapes of spongy mesophyll in the transgenic lines gave rise to lowered area exposed to airspace, which also affected tortuosity. Together, this suggests that the altered cellular architecture in the transgenic lines affects the physiological properties of the leaf.

It is important to note that our conclusions are based on transgenic overexpression of RIC and ROP, which we used as a tool to perturb mesophyll architecture. While our data demonstrate that cytoskeletal regulation can remodel cell and air space geometry, our study does not imply that these overexpression effects fully represent how mesophyll shape is controlled in WT plants. Rather, they provide a tractable system to reveal how targeted perturbations of cytoskeletal pathways can alter leaf internal architecture and thereby impact photosynthetic performance. Future work could build on these findings by leveraging emerging single‐cell RNA expression atlases (Lee *et al*., 2025; Wang *et al*., 2025) to determine whether ROP GTPase pathways are expressed in mesophyll tissue, and to assess their potential role in determining cell polarity inside the leaves of WT plants.

Our findings complement earlier work establishing the critical role of mesophyll porosity, tortuosity, and cell wall thickness in determining mesophyll conductance and photosynthetic performance (Terashima *et al*., 2011; Evans, 2021). Previous work has shown that variation in leaf anatomy can affect diffusional constraints on leaf photosynthesis (Tholen *et al*., 2012), but until recently, genetic mechanisms controlling leaf anatomy remained unclear. Our study addresses this knowledge gap by providing direct evidence that cytoskeletal regulation can remodel mesophyll architecture in ways that influence gas exchange.

In conclusion, we identified a molecular mechanism, the GTPase signalling pathway, which influences internal air space development and impacts the physiological properties of leaves, including cell density and patterning. This pathway underpins significant changes in photosynthetic capacity. By integrating advanced imaging, deep learning, and topological analysis with gas exchange measurements, our study contributes to a new methodological framework for linking cellular structure to leaf function. Future research could build on these findings by exploring how diverse genetic or environmental factors reshape mesophyll architecture to optimise photosynthesis, offering potential applications in crop improvement and climate adaptation strategies.

## Competing interests

None declared.

## Author contributions

IØ and SP designed the research. IØ, AL, GTR, SE and YW collected the data. IØ, SØ, AL and SP performed the research and data analysis. IØ, SØ, AL, JK, ZN and SP wrote the original draft. All authors contributed to the editing of the final manuscript.

## Disclaimer

The New Phytologist Foundation remains neutral with regard to jurisdictional claims in maps and in any institutional affiliations.

## Supporting information


**Fig. S1** Illustration showing persistence homology features 0 and 2 together with binary image representation of 3D data.
**Fig. S2** Illustration of leaf area over time. Each time point shows all measurements (fainted dots) together with average value and SD (error bars).
**Fig. S3** Illustration of all 3D calculations for the three *Arabidopsis thaliana* lines investigated; WT (WT), 35S:RIC1 (RIC) and CA:ROP2 (ROP).
**Fig. S4** Illustrating distributions of the individual palisade cells that are proximal to the adaxial epidermis layer for WT *Arabidopsis thaliana*.
**Fig. S5** Illustration of the two modes for each group of leaf type as well as age.
**Fig. S6** Illustration of the adaxial and abaxial pavement cell layers for the three *Arabidopsis thaliana* lines investigated.
**Fig. S7** Illustration of distributions of the individual palisade cells that are proximal to the adaxial epidermis layer.
**Fig. S8** Illustration of distributions of the individual palisade cells that are proximal to the adaxial epidermis layer.
**Fig. S9** Illustration of the two modes for each group of leaf type as well as age for the three *Arabidopsis thaliana* lines investigated; WT (Col 0), 35S:RIC1 (RIC) and CA:ROP2 (ROP).
**Fig. S10** Illustration of spongy mesophyll cell layer for the three *Arabidopsis thaliana* lines investigated.
**Fig. S11** Illustration of stomata density on 3 and 5 wk old leaves for the three *Arabidopsis thaliana* lines investigated; WT (WT), 35S:RIC1 (RIC) and CA:ROP2 (ROP).
**Fig. S12** Illustration of stomata size on 3 and 5 wk old leaves for the three *Arabidopsis thaliana* lines investigated; WT (WT), 35S:RIC1 (RIC) and CA:ROP2 (ROP).
**Fig. S13** Illustration of mesophyll conductance and net assimilation rate changes upon not assuming a constant and equal photorespiratory compensation point against all lines.
**Fig. S14** Illustration of the stomatal conductance for the three *Arabidopsis thaliana* lines investigated; WT (WT), 35S:RIC1 (RIC) and CA:ROP2 (ROP).
**Fig. S15** Illustration of the net assimilation rate.
**Fig. S16** Illustration of maximal Rubisco carboxylation capacity against the three *Arabidopsis thaliana* lines investigated; WT (WT), 35S:RIC1 (RIC) and CA:ROP2 (ROP).
**Notes S1** Segmentation models.
**Notes S2** Additional measurements and derived photosynthetic parameters over photosynthetic properties between transgenic and WT lines.
**Table S1** Table of ratio of maximum free cell perimeter to cell surface value and SE for the mean of each distribution pooled by age and plant line.
**Table S2** Table of maximum porosity value and SE for the mean of each porosity distribution pooled by age and plant line.
**Table S3** Table of mean palisade length divided into line and age groups.
**Table S4** Table of modes of cell radius of the two first spongy mesophyll cell layers from the abaxial side of the leaf.
**Table S5** Table of modes of radius of internal air space pores around the two first spongy mesophyll cell layers from the abaxial side of the leaf.
**Table S6** Table of maximum tortuosity value for the mean of each porosity distribution pooled by age and plant line.
**Table S7** Table of stomatal density, calculated as stomatal count normalised with scanned leaf surface for 5‐wk‐old leaves on the abaxial pavement layer.
**Table S8** Table of stomatal density and stomata size for leaves used in gas exchange experiments. Shown for the three *Arabidopsis thaliana* lines investigated; WT (WT), 35S:RIC1 (RIC) and CA:ROP2 (ROP).
**Table S9** Table of stomatal density and stomata size.
Please note: Wiley is not responsible for the content or functionality of any Supporting Information supplied by the authors. Any queries (other than missing material) should be directed to the *New Phytologist* Central Office.

## Data Availability

The data segmentation masks and difussion maps that support the findings of this study are openly available in ERDA at (Østerlund & Nyboe Ørting, 2025a,b,c). The Locus IDs corresponding to the transgenic lines used in this study are the following: 35S:RIC1 (AT2G33460) and CA:ROP2 (AT1G20090). The code for segmentation and data analysis is shared in the GitHub repository https://github.com/Oesterlund/arabidopsis‐mesophyll‐airspace.
