# Supplementary material for: Rho GTPase overexpression impacts leaf internal architecture and mesophyll conductance in Arabidopsis
Source: New Phytol. 2025 Oct 13;248(6):3053–66. doi: 10.1111/nph.70618 (PMC12630453; doi:10.1111/nph.70618)
Supplement: Supplementary file 1 — Fig. S1 Illustration showing persistence homology features 0 and 2 together with binary image representation of 3D data. Fig. S2 Illustration of leaf area over time. Each time point shows all measurements (fainted dots) together with average value and SD (error bars). Fig. S3 Illustration of all 3D calculations for the three Arabidopsis thaliana lines investigated; WT (WT), 35S:RIC1 (RIC) and CA:ROP2 (ROP). Fig. S4 Illustrating distributions of the individual palisade cells that are proximal to the adaxial epidermis layer for WT Arabidopsis thaliana. Fig. S5 Illustration of the two modes for each group of leaf type as well as age. Fig. S6 Illustration of the adaxial and abaxial pavement cell layers for the three Arabidopsis thaliana lines investigated. Fig. S7 Illustration of distributions of the individual palisade cells that are proximal to the adaxial epidermis layer. Fig. S8 Illustration of distributions of the individual palisade cells that are proximal to the adaxial epidermis layer. Fig. S9 Illustration of the two modes for each group of leaf type as well as age for the three Arabidopsis thaliana lines investigated; WT (Col 0), 35S:RIC1 (RIC) and CA:ROP2 (ROP). Fig. S10 Illustration of spongy mesophyll cell layer for the three Arabidopsis thaliana lines investigated. Fig. S11 Illustration of stomata density on 3 and 5 wk old leaves for the three Arabidopsis thaliana lines investigated; WT (WT), 35S:RIC1 (RIC) and CA:ROP2 (ROP). Fig. S12 Illustration of stomata size on 3 and 5 wk old leaves for the three Arabidopsis thaliana lines investigated; WT (WT), 35S:RIC1 (RIC) and CA:ROP2 (ROP). Fig. S13 Illustration of mesophyll conductance and net assimilation rate changes upon not assuming a constant and equal photorespiratory compensation point against all lines. Fig. S14 Illustration of the stomatal conductance for the three Arabidopsis thaliana lines investigated; WT (WT), 35S:RIC1 (RIC) and CA:ROP2 (ROP). Fig. S15 Illustration of the net assimilation rate. Fig. [file NPH-248-3053-s001.pdf]

New Phytologist Supporting Information

Article title:

Rho GTPase overexpression impacts leaf internal architecture and mesophyll conductance in Arabidopsis

Authors:

Isabella Østerlund, Silas Ørting, Alistair Leverett, Guillaume Théroux-Rancourt, Samira Ebrahimi, Yang Wang, Zoran Nikoloski, Johannes Kromdijk, Staffan Persson

Article acceptance date:

7 September 2025

This file includes:

Notes S1 - Segmentation models
Notes S2 - Additional measurements and derived photosynthetic parameters over photosynthetic properties between transgenic and WT lines
Figures: S1 Illustration showing persistence homology features 0 and 2 together with binary image representation of 3D data.
S2 Illustration of leaf area over time. Each time point shows all measurements (fainted dots) together with average value and standard deviation (error bars).
S3 Illustration of all 3D calculations for the three *Arabidopsis thaliana* lines investigated; WT (WT), 35S:RIC1 (RIC) and CA:ROP2 (ROP)
S4 Illustrating distributions of the individual palisade cells that are proximal to the adaxial epidermis layer for WT *Arabidopsis thaliana*
S5 Illustration of the two modes for each group of leaf type as well as age.
S6 Illustration of the adaxial and abaxial pavement cell layers for the three *Arabidopsis thaliana* lines investigated.
S7 Illustration of distributions of the individual palisade cells that are proximal to the adaxial epidermis layer.
S8 Illustration of distributions of the individual palisade cells that are proximal to the adaxial epidermis layer.
S9 Illustration of the two modes for each group of leaf type as well as age for the three *Arabidopsis thaliana* lines investigated; WT (Col 0), 35S:RIC1 (RIC) and CA:ROP2 (ROP).

S10 Illustration of spongy mesophyll cell layer for the three *Arabidopsis thaliana* lines investigated.

S11 Illustration of stomata density on 3 and 5 week old leaves for the three *Arabidopsis thaliana* lines investigated; WT (WT), 35S:RIC1 (RIC) and CA:ROP2 (ROP).

S12 Illustration of stomata size on 3 and 5 week old leaves for the three *Arabidopsis thaliana* lines investigated; WT (WT), 35S:RIC1 (RIC) and CA:ROP2 (ROP).

S13 Illustration of mesophyll conductance and net assimilation rate changes upon not assuming a constant and equal photorespiratory compensation point against all lines.

S14 Illustration of the stomatal conductance for the three *Arabidopsis thaliana* lines investigated; WT (WT), 35S:RIC1 (RIC) and CA:ROP2 (ROP).

S15 Illustration of the net assimilation rate.

S16 Illustration of maximal Rubisco carboxylation capacity against the three *Arabidopsis thaliana* lines investigated; WT (WT), 35S:RIC1 (RIC) and CA:ROP2 (ROP).

Tables S1 Table of ratio of maximum free cell perimeter to cell surface value and standard error for the mean of each distribution pooled by age and plant line.

S2 Table of maximum porosity value and standard error for the mean of each porosity distribution pooled by age and plant line.

S3 Table of mean palisade length divided into line and age groups.

S4 Table of modes of cell radius of the two first spongy mesophyll cell layers from the abaxial side of the leaf.

S5 Table of modes of radius of internal air space pores around the two first spongy mesophyll cell layers from the abaxial side of the leaf.

S6 Table of maximum tortuosity value for the mean of each porosity distribution pooled by age and plant line.

S7 Table of stomatal density, calculated as stomatal count normalised with scanned leaf surface for five-week-old leaves on the abaxial pavement layer.

S8 Table of stomatal density and stomata size for leaves used in gas exchange experiments. Shown for the three *Arabidopsis thaliana* lines investigated; WT (WT), 35S:RIC1 (RIC) and CA:ROP2 (ROP).

S9 Table of stomatal density and stomata size.

Notes S1

All networks were implemented in python using pytorch (Paszke et al. 2019), pytorch-lightning (Falcon & The PyTorch Lightning team 2019) and MONAI (Cardoso et al. 2022). SciPy (Virtanen et al. 2020) and scikit-image (Van Der Walt et al. 2014) were used for post-processing. Architecture details and parameters are provided below and the implementation is available at https://github.com/Oesterlund/arabidopsis-mesophyll-airspace.

Semantic segmentation

A 2D UNet was trained to segment five classes, mesophyll, outside leaf, veins, adaxial pavement, abaxial pavement, and internal air. We used the UNet from MONAI, with parameters `channels = [32, 32, 64, 64, 128, 128, 256]`, `strides = (1, 2, 1, 2, 1, 2)`, and `num_res_units = 3`. We used the Adam optimizer with a multi-label dice loss and a learning rate of 0.001. We augmented the training data by randomly flipping the images with a 50% probability.

Mesophyll instance segmentation

Individual mesophyll cells were segmented using in a multi-step approach. First a 2D UNet was trained to segment cell boundaries. We used the UNet from MONAI, with parameters `channels = [32, 32, 64, 64, 128, 128, 256]`, `strides = (1, 2, 1, 2, 1, 2)`, and `num_res_units = 3`. We used the Adam optimizer with a binary dice loss and a learning rate of 0.001. We used an axis-parallel multi-planar approach to obtain smoother 3D segmentations. We zero-padded the volumes to a size of $640 \times 640 \times 640$ voxels. For training we extracted slices at indices 150, 225, 300, 375, and 450 along all three axes. For prediction we extracted all slices along all three axes and averaged the prediction in each voxel. Initially the network was trained on a small number of slices derived from a watershed segmentation

We then dilated the cell boundary predictions with a ball of radius 5 voxels to close any holes and used connected component analysis to segment individual cells from the inverse of the boundary segmentation. Finally, the segmented cells were expanded up to 10 voxels inside the semantic mesophyll segmentation.

Palisade and spongy mesophyll segmentation

We labeled individual mesophyll cells as palisade or spongy based on their distance to the adaxial and abaxial pavement cells. Any cell within 10 voxels of the adaxial pavement layer was labeled as palisade cells. Any cell within 20 voxels of the abaxial pavement layer was labeled as spongy cells.

Notes S2

Transgenic Lines Lead to Reduced Stomatal Conductance

In addition to exploring differences in mesophyll conductance, we further investigated other aspects of photosynthetic physiology in the two transgenic lines. A difference in net assimilation rate across the range of atmospheric CO_2 concentrations could be attributed altered stomatal conductance (g_{sc}), mesophyll conductance (g_m), or underlying biochemistry. We first explored stomatal conductance, given that Rho-type GTPases and their interactors have been shown to affect stomatal apertures (Jeon et al. 2008; Hong et al. 2016).

We found that both transgenic lines exhibited lower g_{sc} values compared to WT plants, across all CO_2 concentration treatments (SI Figure S14 A). No significant differences in stomatal density were observed in the leaves used for gas exchange (SI Table S8). If alterations in A_n were solely dependent on stomatal conductance, we would expect similar A_n values for similar CO_2 concentrations in the substomatal cavities (c_i) across the three plant lines (Busch et al. 2024). However, A_n was consistently lower in both transgenic lines than in WT for similar c_i values (SI Figure S14 B), demonstrating that variations in g_{sc} alone were insufficient to explain the changes in A_n. Therefore, the remaining differences in A_n must be attributed to reduced mesophyll conductance, biochemical capacity, or a combination of both.

Transgenic lines lead to reduced biochemical capacity for photosynthesis

Having established that both transgenic lines exhibited lower mesophyll conductance than WT (Figure 4 D), we aimed to determine if the alterations in the transgenic lines affected the biochemical capacity for photosynthesis. Due to the inability to construct full A_n/c_i curves from the estimated CO_2 concentration in internal airspace values, we could not estimate the maximum rate of carboxylation or the maximum rate of electron transport, having the limiting factors for net assimilation rate unclear.

We observed that ROP leaves had lower assimilation rates at any given c_c compared to WT, suggesting a reduced biochemical capacity for photosynthesis in the ROP transgenic line. In contrast, RIC showed more similar tendencies to WT (SI Figure S15 B), indicating that the RIC transgenic line did not negatively impact the biochemical capacity for photosynthesis as severely as ROP. These results suggest that the transgenic lines have multiple effects on leaf physiology, including reduced stomatal conductance, mesophyll conductance, and biochemical capacity for photosynthesis. This coincides with the worsened tortuosity (Figure 4 A-B), with ROP being worse than RIC compared to WT, and ROP exhibiting the lowest porosity and air-to-cell surface measures relative to both RIC and WT (Figure 3 A-B).

Robustness of Mesophyll Conductance Estimates

The estimations of mesophyll conductance used in previous derivations and result sections were based on the assumption that the photorespiratory compensation point was constant (42.5 $\mu mol \cdot mol^{-1}$) across all genotypes. To test the robustness of this assumption, we estimated the apparent photorespiratory compensation point (for derivations, please refer to the materials and methods section). Using the new estimations, mesophyll conductance remained significantly lower in the transgenic lines compared to WT, and the net assimilation rate to CO_2 concentration in the chloroplast relationships was consistent with the results using a constant photorespiratory compensation point (SI Figure S13). This result indicates that the lower g_m estimates in RIC and ROP plants were sufficiently robust, whether the photorespiratory compensation point was assumed to be constant or estimated to vary between genotypes.

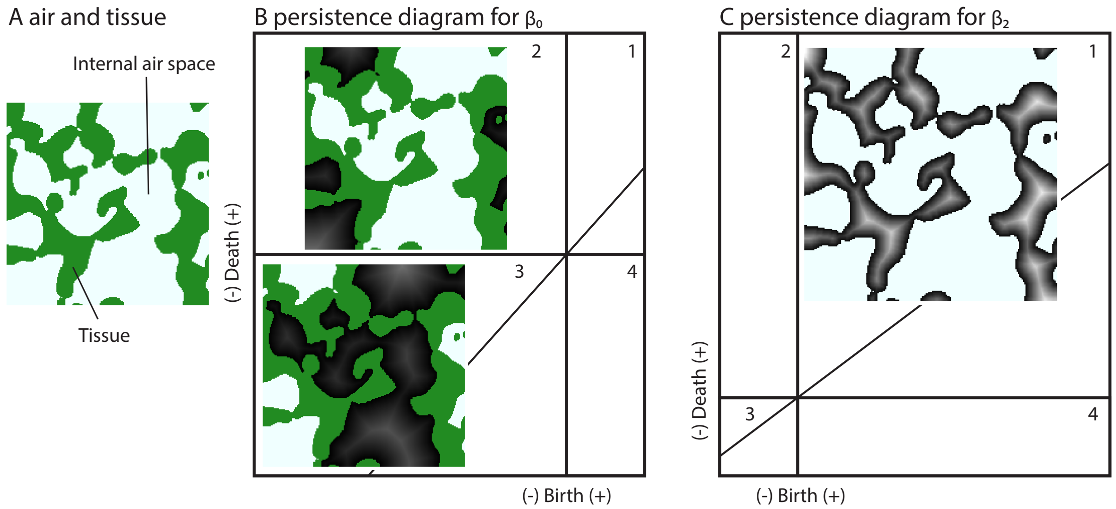

Figure S1: Illustration showing persistence homology features 0 and 2 together with binary image representation of 3D data. Panel **A** illustrate binary 2D image data divided into (green) tissue, representing positive distance values, and (azure), representing negative distance values for internal air space. **B** illustrate a persistence diagram of the birth and death for homology feature 0 together with distance values for the negative distance from tissue space to air space (white is negative largest distance, and black is zero distance). **C** illustrate a persistence diagram of the birth and death for homology feature 2 together with distance values for the positive distance from air space to tissue space (white is positive largest distance, and black is zero distance.

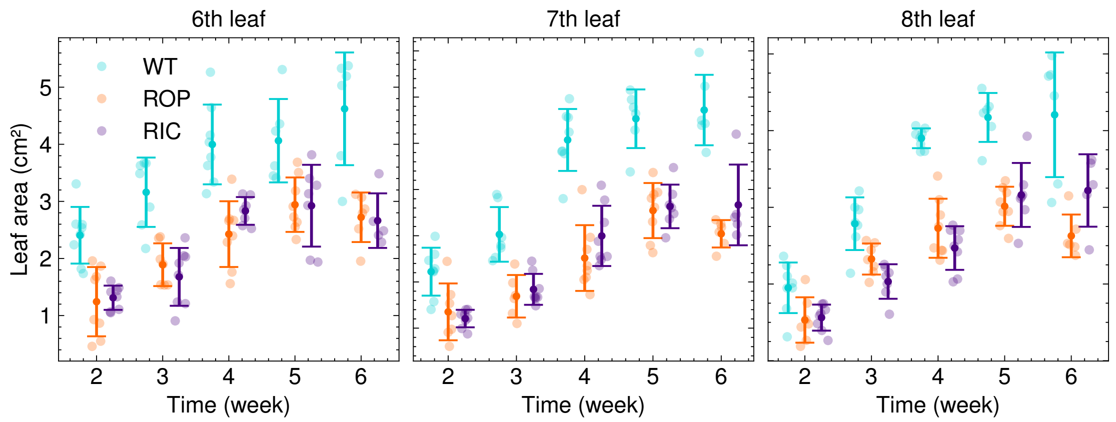

Figure S2: Illustration of leaf area over time. Each time point shows all measurements (fainted dots) together with average value and standard deviation (error bars). The data shown here was acquired as described in the *Stomata density and size calculation for leaf size measurements* subsection.

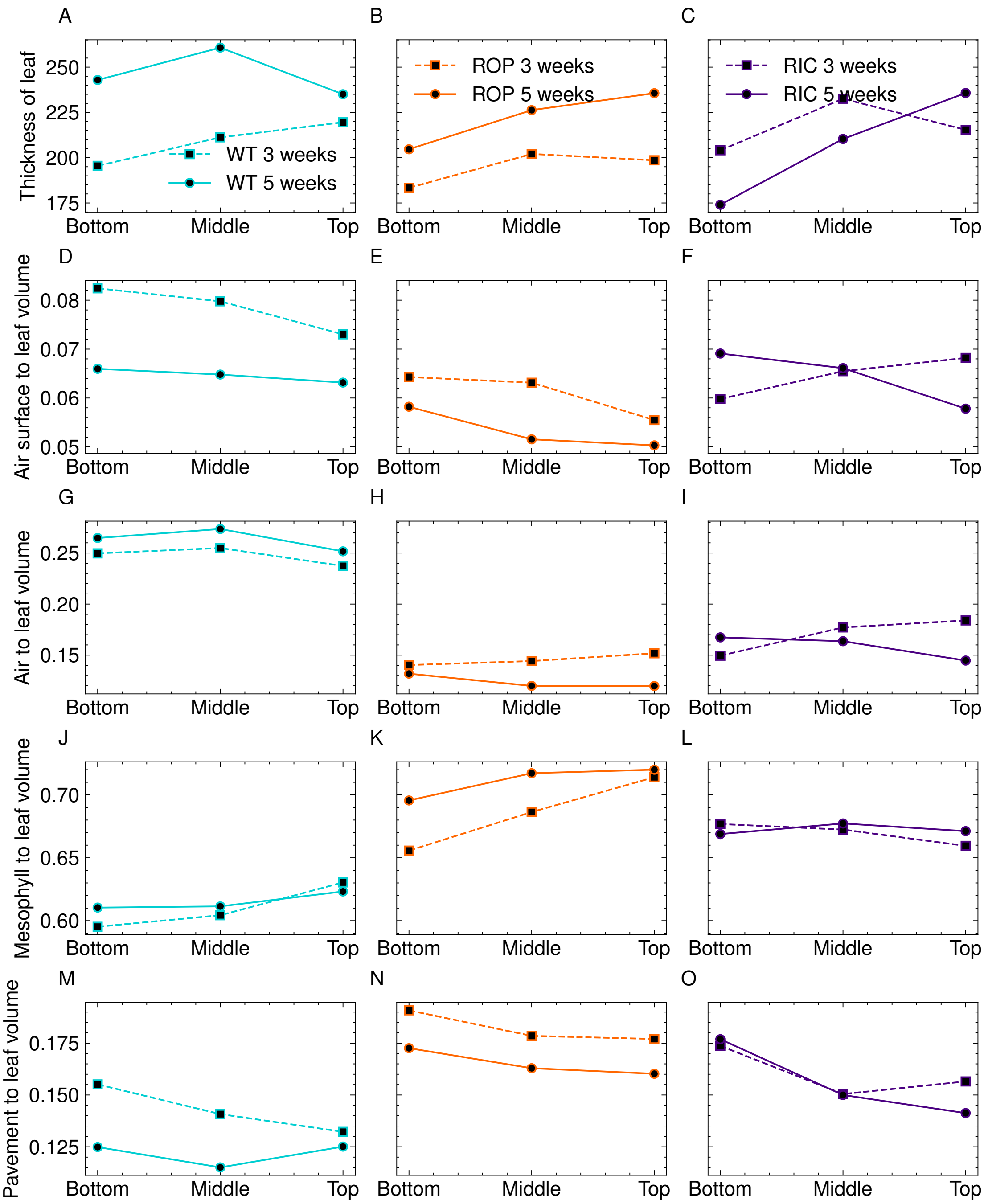

Figure S3: Illustration of all 3D calculations for the three *Arabidopsis thaliana* lines investigated; WT (WT), 35S:RIC1 (RIC) and CA:ROP2 (ROP). **A, B, C** illustrates mean thickness of leaves for both 3 and 5 week old leaves. **D, E, F** illustrate air surface to leaf volume, calculated as the surface of cells touching air to the full volume of the leaf scans. **G,H, I** illustrate air volume to leaf volume. **J, K, L** illustrate both palisade and spongy mesophyll volume to leaf volume. **M, N, O** illustrate the epidermal cell layer volume to leaf volume.

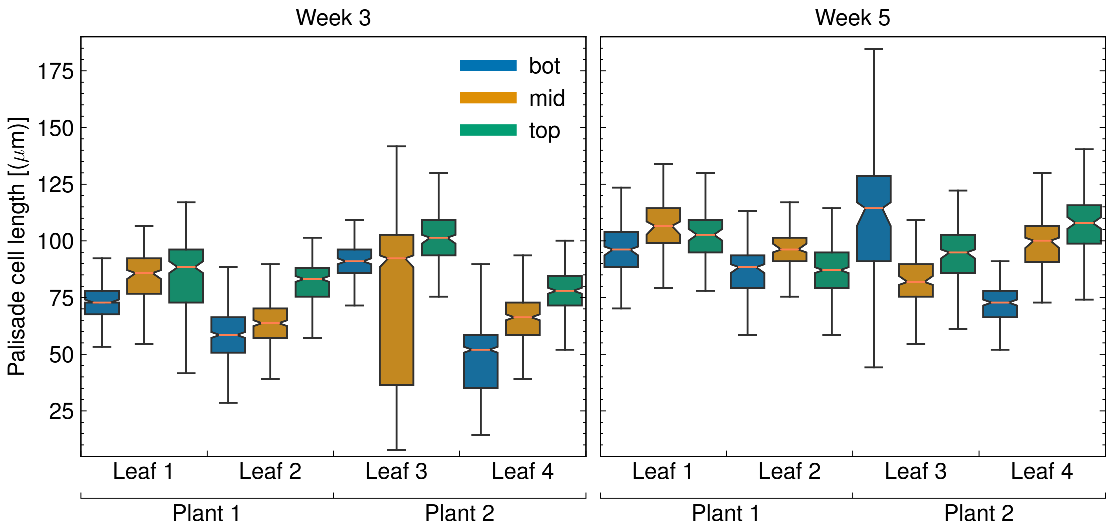

Figure S4: Illustrating distributions of the individual palisade cells that are proximal to the adaxial epidermis layer for WT *Arabidopsis thaliana*. The data is divided into individual scans and positions on each leaf to illustrate the patterns occurring in length changes across the leaves of 3 week- and 5 week-old WT plants. The leaf numbers correspond to different leaves from the same plants, given here that leaf 1 and 2 is from the same plant, plant 1, and leaf 3 and 4 is from the same, but different plant, plant 2. Error bars represent 90% confidence interval, bootstrapped 1000 times.

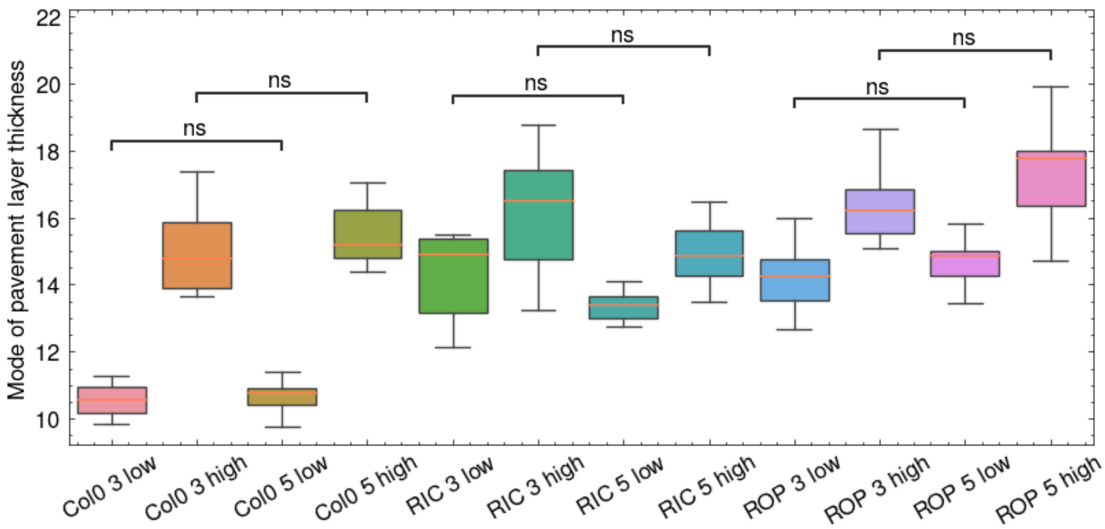

Figure S5: Illustration of the two modes for each group of leaf type as well as age. Each group of leaf type corresponds to the three *Arabidopsis thaliana* lines investigated; WT (Col 0), 35S:RIC1 (RIC) and CA:ROP2 (ROP). The distribution of thickness of the pavement layers followed a bimodal distribution, with one relatively high and one low valued peak. This is captured by the mode of the two peaks in the bimodal distribution. Shown here with Mann–Whitney U test between the corresponding low or high peaks between same genotype. Error bars represent 90% confidence interval, bootstrapped 1000 times.

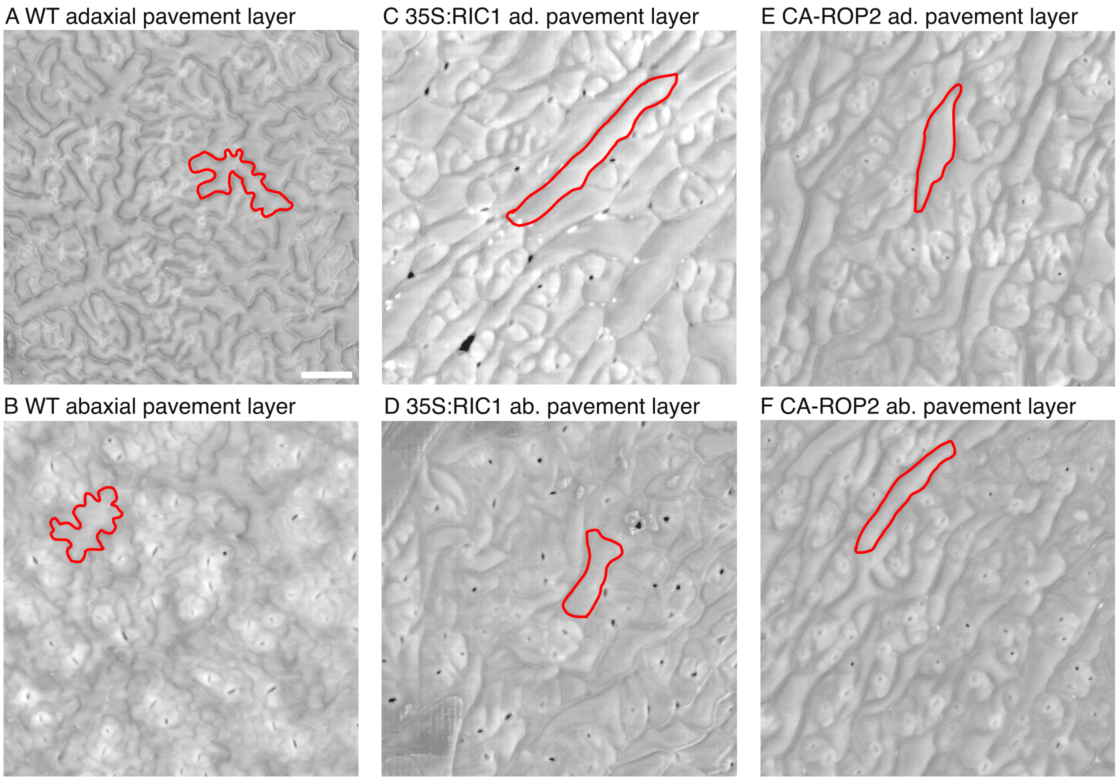

Figure S6: Illustration of the adaxial and abaxial pavement cell layers for the three *Arabidopsis thaliana* lines investigated. **A-B** illustrate the pavement cell layers for WT, **C-D** for 35S:RIC1, and **E-F** for CA:ROP2. In each image, one cell is highlighted in red. All images are 2D max projections of 3D masked pavement image data. The scale bar in panel A is 100 μm, representative of A-F.

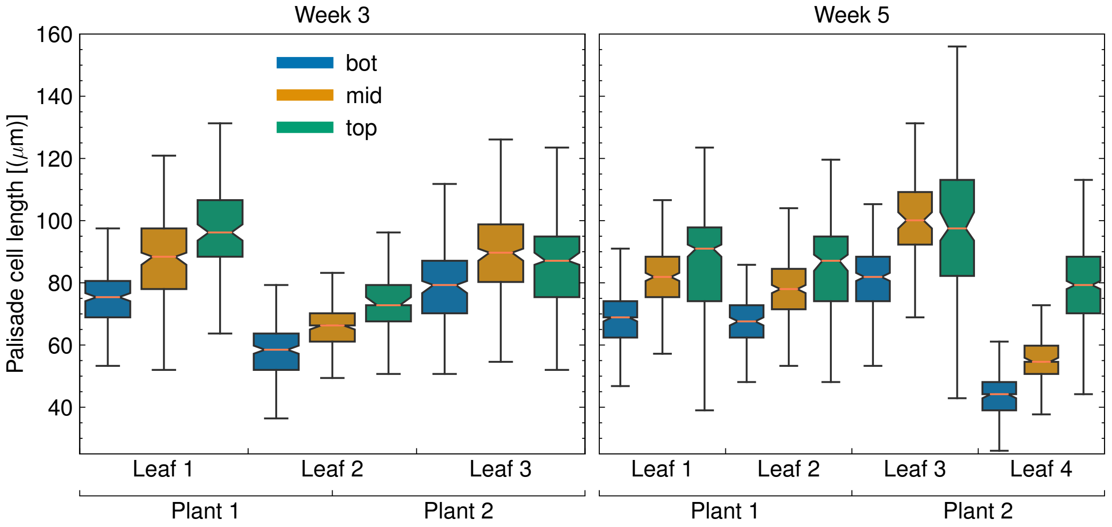

Figure S7: Illustration of distributions of the individual palisade cells that are proximal to the adaxial epidermis layer. The data is divided into individual scans and positions on each leaf to illustrate the patterns occurring in length changes across the leaves of 3 week- and 5 week-old RIC plants. The leaf numbers correspond to different leaves from the same plants, given here that leaf 1 and 2 is from the same plant, plant 1, and leaf 3 is from the same, but different plant, plant 2. Error bars represent 90% confidence interval, bootstrapped 1000 times.

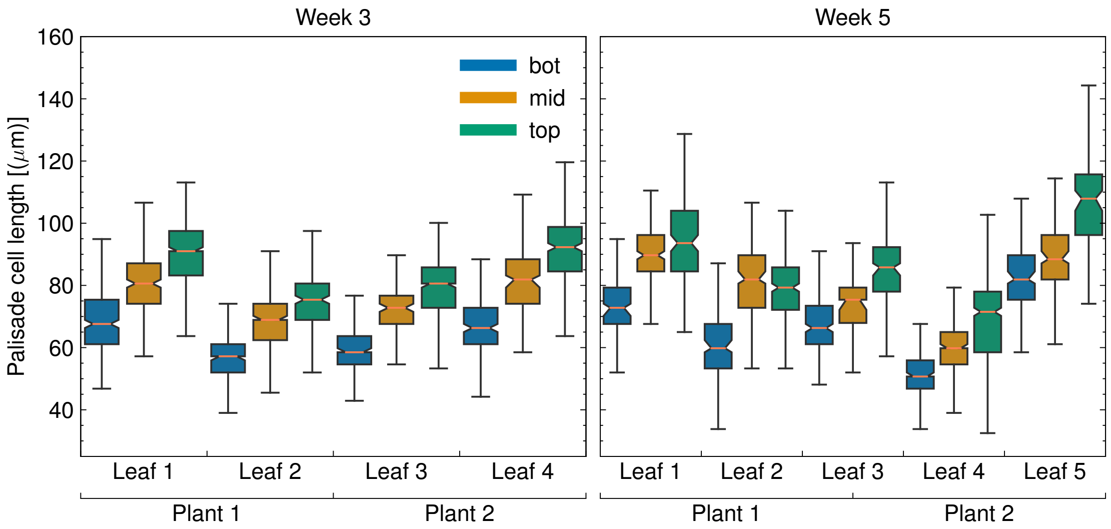

Figure S8: Illustration of distributions of the individual palisade cells that are proximal to the adaxial epidermis layer. The data is divided into individual scans and positions on each leaf to illustrate the patterns occurring in length changes across the leaves of 3 week- and 5 week-old ROP plants. The leaf numbers correspond to different leaves from the same plants, given here that leaf 1 and 2 is from the same plant, plant 1, and leaf 3 and 4 is from the same, but different plant, plant 2. Error bars represent 90% confidence interval, bootstrapped 1000 times.

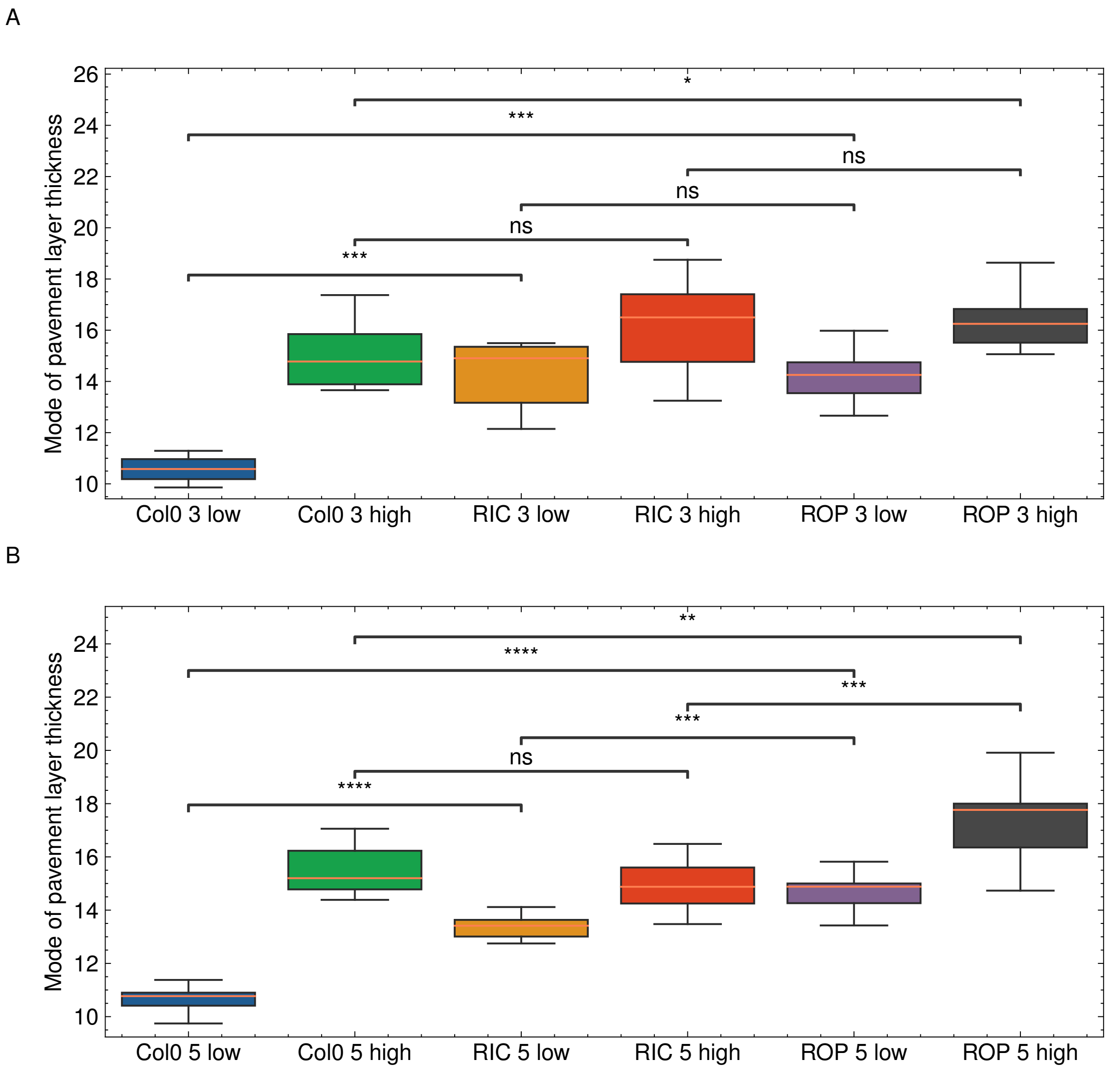

Figure S9: Illustration of the two modes for each group of leaf type as well as age for the three *Arabidopsis thaliana* lines investigated; WT (Col 0), 35S:RIC1 (RIC) and CA:ROP2 (ROP). The distribution of thickness of the pavement layers followed a bimodal distribution, with one relatively high and one low valued peak. This is captured by the mode of the two peaks in the bimodal distribution. **A** illustrate modes for 3 week- and **B** for 5 week-old leaves. Shown here with Mann–Whitney U test between the corresponding low or high peaks between different genotypes. Error bars represent 90% confidence interval, bootstrapped 1000 times.

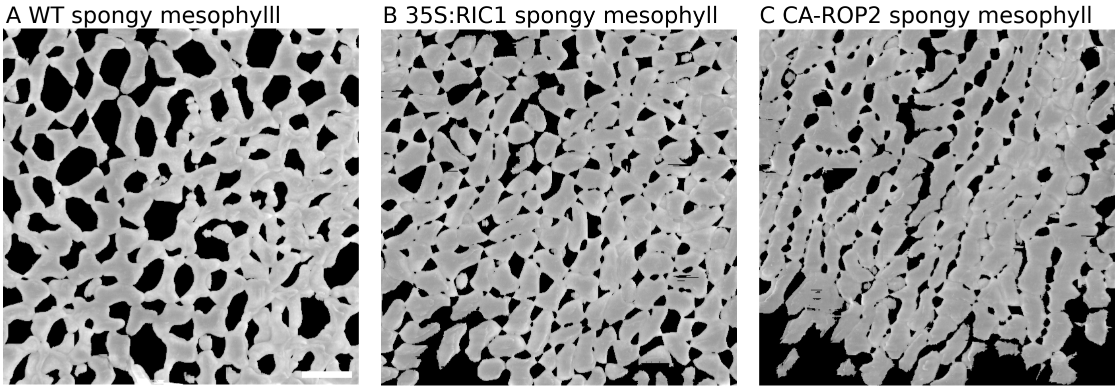

Figure S10: Illustration of spongy mesophyll cell layer for the three *Arabidopsis thaliana* lines investigated. **A** illustrate a spongy mesophyll cell layer for WT, **B** for 35S:RIC1, and **C** for CA:ROP2. All images are 2D max projections of 3D masked spongy mesophyll image data. The scale bar in panel A is 100 μm, representative of A-C.

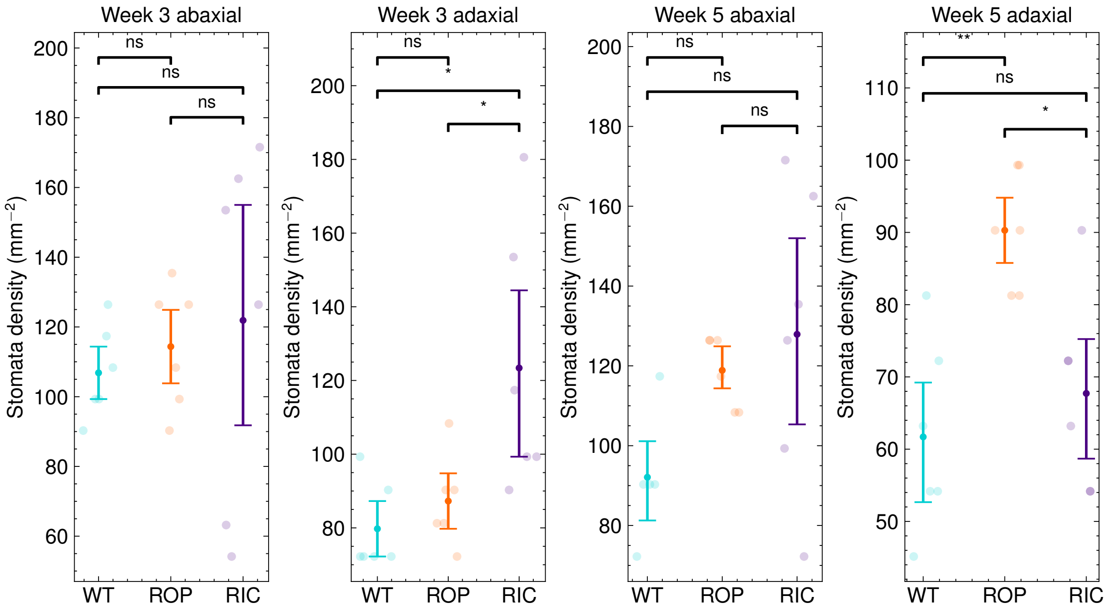

Figure S11: Illustration of stomata density on 3 and 5 week old leaves for the three *Arabidopsis thaliana* lines investigated; WT (WT), 35S:RIC1 (RIC) and CA:ROP2 (ROP). Each category contains 3 different leaves, using the 7th leaf for each genotype. The plots show measurements for both abaxial and adaxial stomata density for week 3 and 5 leaves. Shown with mean and error bars, dots illustrate stomatal density for each scan. Error bars represent 90% confidence interval, bootstrapped 10000 times. p-values were determined using Tukey method. The data shown here was acquired as described in the *Stomata density and size calculation for leaf size measurements* subsection.

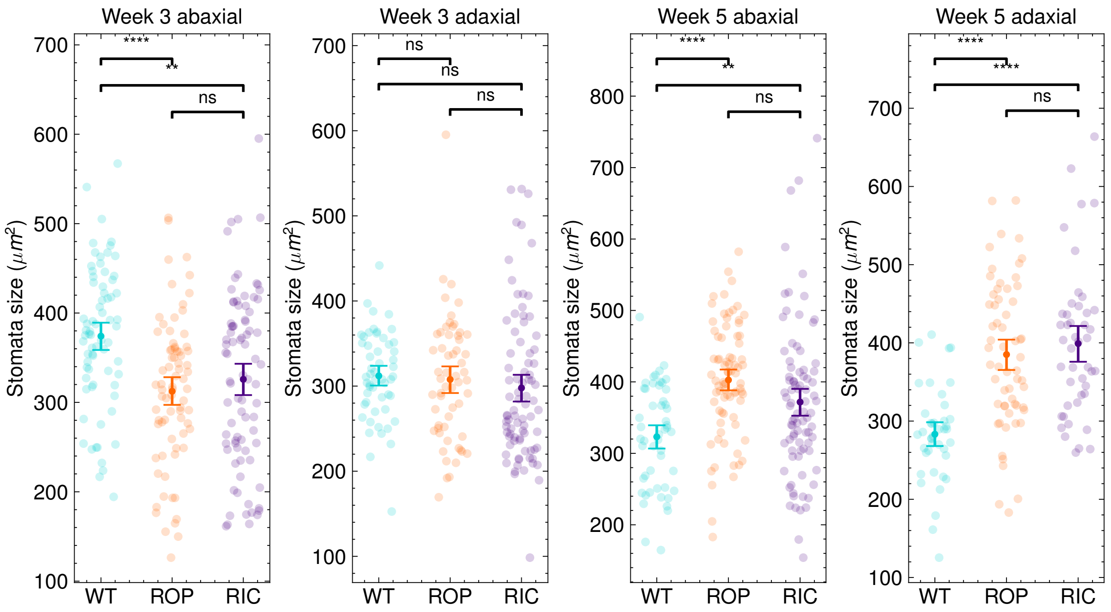

Figure S12: Illustration of stomata size on 3 and 5 week old leaves for the three *Arabidopsis thaliana* lines investigated; WT (WT), 35S:RIC1 (RIC) and CA:ROP2 (ROP). Each category contains 3 different leaves, using the 7th leaf for each genotype. The plots show measurements for both abaxial and adaxial stomata sizes for week 3 and 5 leaves. Shown with mean and error bars, dots illustrate individual measurements. Error bars represent 90% confidence interval, bootstrapped 10000 times. p-values were determined using Tukey method. The data shown here was acquired as described in the *Stomata density and size calculation for leaf size measurements* subsection.

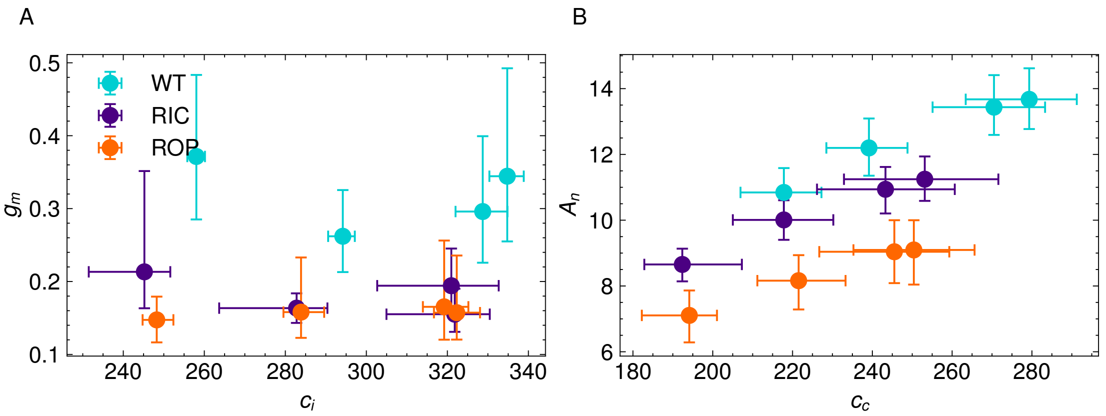

Figure S13: Illustration of mesophyll conductance and net assimilation rate changes upon not assuming a constant and equal photorespiratory compensation point against all lines. Shown for the three *Arabidopsis thaliana* lines investigated; WT (WT), 35S:RIC1 (RIC) and CA:ROP2 (ROP). **A** illustrate mesophyll conductance against CO_2 concentration in internal air space. **B** illustrate net assimilation rate against chloroplast CO_2 concentration. Error bars represent 90% confidence interval, bootstrapped 1000 times.

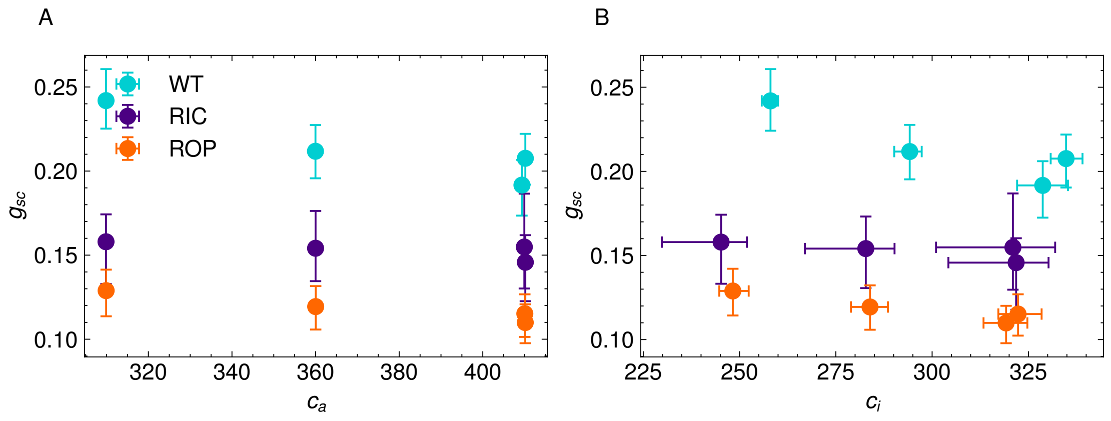

Figure S14: Illustration of the stomatal conductance for the three *Arabidopsis thaliana* lines investigated; WT (WT), 35S:RIC1 (RIC) and CA:ROP2 (ROP). **A** illustrate stomatal conductance against atmospheric CO_2 concentrations. **B** illustrate stomatal conductance against CO_2 concentration in internal air space. All values were measured or derived from steady-state values. Error bars represent 90% confidence interval, bootstrapped 1000 times.

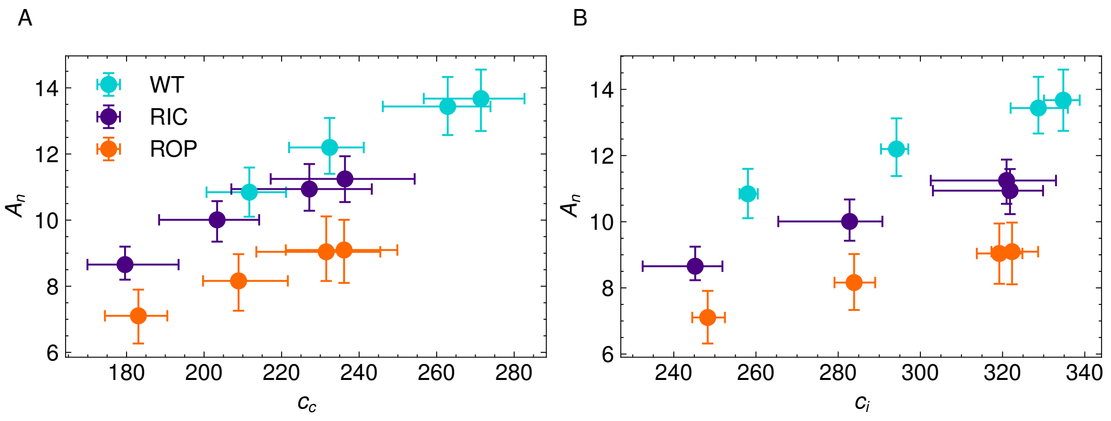

Figure S15: Illustration of the net assimilation rate. **A** illustrate net assimilation rate against chloroplast CO_2 concentrations. **B** illustrate net assimilation rate against CO_2 concentration in internal air space. Error bars represent 90% confidence interval, bootstrapped 1000 times.

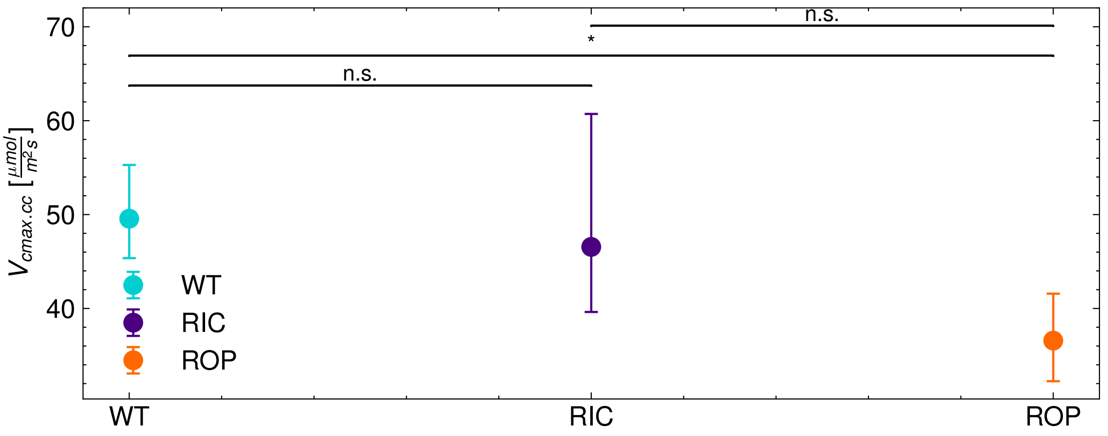

Figure S16: Illustration of maximal Rubisco carboxylation capacity against the three *Arabidopsis thaliana* lines investigated; WT (WT), 35S:RIC1 (RIC) and CA:ROP2 (ROP). A Kruskal-Wallis p-test was performed on full data giving p-value: 0.037. The statistical test shown are Dunn's post-hoc test. Error bars represent 90% confidence interval, bootstrapped 1000 times.

Line	Maximum free cell surface 3 weeks	Standard deviation	Maximum free cell surface 5 weeks	Standard deviation
WT	0.13	0.017	0.13	0.011
RIC	0.11	0.012	0.10	0.016
ROP	0.093	0.011	0.084	0.010

Table S1: Table of ratio of maximum free cell perimeter to cell surface value and standard error for the mean of each distribution pooled by age and plant line. Shown for the three *Arabidopsis thaliana* lines investigated; WT (WT), 35S:RIC1 (RIC) and CA:ROP2 (ROP). The values are unit less, as they are ratios.

Line	Maximum porosity 3 weeks	Standard error	Maximum porosity 5 weeks	Standard deviation
WT	0.42	0.04	0.50	0.04
RIC	0.31	0.04	0.27	0.04
ROP	0.22	0.03	0.22	0.03

Table S2: Table of maximum porosity value and standard error for the mean of each porosity distribution pooled by age and plant line. Shown for the three *Arabidopsis thaliana* lines investigated; WT (WT), 35S:RIC1 (RIC) and CA:ROP2 (ROP). Based on Welch t-test nearly all group pairs differed significantly (p-val < 0.05), with very strong differences between them. Only ROP W3 to W5 did not show a significant difference.

Line	Mean palisade length [μm]	Standard error
WT 3 weeks	58.5	0.29
WT 5 weeks	71.9	0.51
RIC 3 weeks	70.5	0.36
RIC 5 weeks	64.1	0.29
ROP 3 weeks	64.4	0.25
ROP 5 weeks	67.2	0.30

Table S3: Table of mean palisade length divided into line and age groups. Shown for the three *Arabidopsis thaliana* lines investigated; WT (WT), 35S:RIC1 (RIC) and CA:ROP2 (ROP). The standard error is bootstrapped 1000 times.

Line	Mode [μm] 3 weeks	Standard deviation	Mode [μm] 5 weeks	Standard deviation
WT	14.04	2.07	15.41	1.47
RIC	17.74	2.72	16.97	3.02
ROP	15.69	2.01	16.62	1.89

Table S4: Table of modes of cell radius of the two first spongy mesophyll cell layers from the abaxial side of the leaf. Shown for the three *Arabidopsis thaliana* lines investigated; WT (WT), 35S:RIC1 (RIC) and CA:ROP2 (ROP). Shown with standard deviation on modes. The modes and standard deviations are found based on individual scans fitted Gaussian based kernel density estimates.

Line	Mode [μm] 3 weeks	Standard deviation	Mode [μm] 5 weeks	Standard deviation
WT	9.82	2.77	11.38	2.43
RIC	5.81	1.81	4.88	0.91
ROP	4.55	0.89	4.16	0.62

Table S5: Table of modes of radius of internal air space pores around the two first spongy mesophyll cell layers from the abaxial side of the leaf. Shown for the three *Arabidopsis thaliana* lines investigated; WT (WT), 35S:RIC1 (RIC) and CA:ROP2 (ROP). Shown with standard deviation on modes. The modes and standard deviations are found based on individual scans fitted Gaussian based kernel density estimates.

Line	Maximum tortuosity 3 weeks	Maximum tortuosity 5 weeks
WT	1.04	1.04
RIC	1.09	1.11
ROP	1.12	1.14

Table S6: Table of maximum tortuosity value for the mean of each porosity distribution pooled by age and plant line. Shown for the three *Arabidopsis thaliana* lines investigated; WT (WT), 35S:RIC1 (RIC) and CA:ROP2 (ROP).

Line<br>Abaxial	Stomatal density [mm^{-2}]	90% CI x10^{-6}
WT 5 weeks	146	124, 162]
RIC 5 weeks	221	[172, 311]
ROP 5 weeks	262	[232, 298]

Table S7: Table of stomatal density, calculated as stomatal count normalised with scanned leaf surface for five-week-old leaves on the abaxial pavement layer. Shown for the three *Arabidopsis thaliana* lines investigated; WT (WT), 35S:RIC1 (RIC) and CA:ROP2 (ROP). The confidence interval is bootstrapped 1000 times.

Line<br>Abaxial	Stomatal density<br>$[mm^{-2}]$	90% CI x10^{-6}	Stomatal size<br>$[\mu m^2]$	90% CI
WT 10 weeks	0.66	[0.55, 0.89]	27754.2	[26471.8, 29790.4]
RIC 10 weeks	0.52	[0.45, 0.58]	23465.2	[21899.4, 25609.5]
ROP 10 weeks	0.40	[0.33, 0.47]	34655.1	[32389.9, 36455.4]

Table S8: Table of stomatal density and stomata size for leaves used in gas exchange experiments. Shown for the three *Arabidopsis thaliana* lines investigated; WT (WT), 35S:RIC1 (RIC) and CA:ROP2 (ROP). Stomatal density was calculated as stomatal count normalised with leaf surface for approximately ten-week-old leaves on the abaxial pavement layer. The confidence interval is bootstrapped 1000 times.

Line<br>Adaxial	Stomatal density<br>$[mm^{-2}]$	90% CI x10^{-6}	Stomatal size<br>$[\mu m^2]$	90% CI
WT 5 weeks	158	[143, 172]	303.5	[290.2, 328.5]
RIC 5 weeks	208	[145, 282]	364.0	[341.2, 379.1]
ROP 5 weeks	266	[241, 301]	376.3	[364.3, 390.3

Table S9: Table of stomatal density and stomata size. Density is calculated as stomatal count normalised with scanned leaf surface for five-week-old leaves on the adaxial pavement layer. Shown for the three *Arabidopsis thaliana* lines investigated; WT (WT), 35S:RIC1 (RIC) and CA:ROP2 (ROP). Stomatal size is calculated as stomatal coverage averaged per image stack, averaged over all image data. The confidence interval is bootstrapped 1000 times.

References

Jeon, B. W. et al. (Feb. 2008). "The Arabidopsis Small G Protein ROP2 Is Activated by Light in Guard Cells and Inhibits Light-Induced Stomatal Opening". In: *The Plant Cell* 20.1, pp. 75–87. ISSN: 1532-298X. DOI: 10.1105/tpc.107.054544.

Van Der Walt, S., J. L. Schönberger, J. Nunez-Iglesias, F. Boulogne, J. D. Warner, N. Yager, E. Gouillart & T. Yu (2014). "Scikit-image: Image processing in python". In: *PeerJ* 2014.1. ISSN: 21678359. DOI: 10.7717/peerj.453.

Hong, D., B. W. Jeon, S. Y. Kim, J.-U. Hwang & Y. Lee (Jan. 2016). "The ROP2-RIC7 pathway negatively regulates light-induced stomatal opening by inhibiting exocyst subunit Exo70B1 in Arabidopsis". In: *New Phytologist* 209.2, pp. 624–635. ISSN: 0028-646X. DOI: 10.1111/nph.13625.

Falcon, W. & The PyTorch Lightning team (Mar. 2019). *PyTorch Lightning*. DOI: 10.5281/zenodo.3828935. URL: https://github.com/Lightning-AI/lightning.

Paszke, A. et al. (2019). "PyTorch: An Imperative Style, High-Performance Deep Learning Library". In: *Advances in Neural Information Processing Systems 32*. Ed. by H. Wallach, H. Larochelle, A. Beygelzimer, F. d Alché-Buc, E. Fox & R. Garnett. Curran Associates, Inc., pp. 8024–8035. URL: http://papers.neurips.cc/paper/9015-pytorch-an-imperative-style-high-performance-deep-learning-library.pdf.

Virtanen, P. et al. (Mar. 2020). "SciPy 1.0: fundamental algorithms for scientific computing in Python". In: *Nature Methods* 17.3, pp. 261–272. ISSN: 15487105. DOI: 10.1038/s41592-019-0686-2.

Cardoso, M. J. et al. (Nov. 2022). "MONAI: An open-source framework for deep learning in healthcare". In: *arXiv*.

Busch, F. A. et al. (Feb. 2024). "A guide to photosynthetic gas exchange measurements: Fundamental principles, best practice and potential pitfalls". In: *Plant, Cell & Environment*. ISSN: 0140-7791. DOI: 10.1111/pce.14815.
